# Assessing clenbuterol’s modulation of metabolic and inflammatory pathways in Nile tilapia (*Oreochromas niloticous*) fed high fat diet

**DOI:** 10.1038/s41598-024-84814-1

**Published:** 2025-01-10

**Authors:** Aya G. Rashwan, Doaa H. Assar, Abdallah S. Salah, Muyassar H. Abualreesh, Shimaa M. R. Salem, Norah Althobaiti, Zizy I. Elbialy

**Affiliations:** 1 Faculty of Aquatic and Fisheries Sciences, Kafrelsheikh University, Kafrelsheikh, 33516 Egypt; 2 Clinical Pathology Department, Faculty of Veterinary Medicine, Kafrelsheikh University, Kafrelsheikh, 33516 Egypt; 3 Institute of Aquaculture, Faculty of Natural Sciences, University of Stirling, Stirling, FK9 4LA UK; 4 Department of Marine Biology, Faculty of Marine Sciences, King Abdul-Aziz University (KAU), 21589 Jeddah, Saudi Arabia; 5 Center of Excellence for Environmental Studies (CEES), King Abdulaziz University, 22252 Jeddah, Saudi Arabia; 6 Department of Animal Nutrition and Nutritional Deficiency Diseases, Faculty of Veterinary Medicine, Mansoura University, Mansoura, Egypt; 7 Biology Department, College of Science and Humanities-Al Quwaiiyah, Shaqra University, 19257 Al Quwaiiyah, Saudi Arabia

**Keywords:** Biological techniques, Molecular biology, Physiology

## Abstract

This study was performed to reveal the metabolic effects and molecular mechanisms that govern the dietary incorporation of clenbuterol on growth performance, haemato-biochemical changes, histological alteration, and gene expression regulating glucose and lipid metabolism in normal and high-fat diets fed in Nile tilapia (*Oreochromis niloticus*). Six experimental diets were formulated, incorporating different concentrations of clenbuterol. The 1st three groups were supplemented with a diet comprising 6% fat, with clenbuterol of 0, 5, and 10 g/kg diet was designated as F6 clenb0, F6clenb5, and F6clenb10, respectively. The other treatment groups were fed a diet of 12% fat, with clenbuterol 0, 5, and 10 g/kg diet, respectively termed F12 clenb0, F12 clenb5, and F12 clenb10. The results revealed that compared to the control group, HFD exhibited a marked reduction in FBW, BWG, PER, and body protein percent but significantly increased the FCR, IPF, liver fat percent, and body ash percent with altered hematological parameters, raised serum biomarkers of hepatic and renal injury. HFD signally raised mRNA expression of pro-inflammatory cytokines, and declined *nrf2* and antioxidative function-related genes. Also increased mRNA expression of lipogenic genes as FAS and SREBP-1c and gluconeogenic genes as *pepck* and *g6pc* while downregulated, *pparα*, *cpt1*, *acox1*. Nevertheless, clenbuterol supplementation significantly reversed the aforementioned findings induced by HFD. Clenbuterol inclusion significantly improves growth performance and antioxidant defenses by modulating *nrf2* signaling and reducing inflammatory response, reduces fatty acid synthesis, and enhances mitochondrial β-oxidation not only functioning as a lipid regulator and effectively alleviating fat accumulation in the liver but playing an essential role in the control of glucose metabolism by reducing hepatic glucose production in high-fat diet-fed Nile tilapias well.

## Introduction

The passage emphasizes the significance of lipids as fundamental nutrients for aquaculture species, serving as a source of energy and vital fatty acids necessary for regular physiological activities. It is essential to fine-tune the lipid content in the diet to ensure the production of high-quality end products^1,2^. Nevertheless, an overabundance of lipids can adversely affect the development and well-being of fish, resulting in reduced feed consumption, slower growth rates, weakened immune response, and increased oxidative stress^3–6^. This data derives from research conducted on several fish species, including black seabream (*Acanthopagrus schlegelii*), the large yellow croaker (*Larmichthys crocea*), and blunt snout bream (*Megalobrama amblycephala*)^7,8^. Four distinct processes can lead to the accumulation of lipids in the liver: enhanced absorption of circulating fatty acids by the liver, promoted synthesis of new fatty acids within the liver, diminished beta-oxidation in the liver, and reduced export of lipids from the liver^9^. SREBP-1c stimulates genes associated with fatty acid and triglyceride synthesis, as fatty acid synthase (FAS)^10,11^. Carnitine palmitoyltransferase-1 (CPT-1) acts as a crucial regulator, controlling the entry of fatty acids into the mitochondria, which is critical for the β-oxidation of fatty acids^12^. Diacylglycerol acyltransferase DGAT2 is critical in lipid accumulation as it catalyzes the last step in the synthesis of triacylglycerol (TAG)^13,14^. Additionally, the liver controls gluconeogenesis, where enzymes such as glucose-6-phosphatase (G6PC) and phosphoenolpyruvate carboxylase kinase (PEPCK) play crucial roles, leading to heightened production of glucose by the liver^15,16^.

Recent research has shown that various dietary supplements are effective in managing lipid metabolism, which helps in reducing fat buildup, easing hepatic steatosis, and diminishing inflammatory reactions^17–19^. Feed supplements, such as β2-agonists, are used to increase the proportion of lean meat in carcasses and decrease the feed needed for animals with higher muscle-to-fat ratios^20^. Clenbuterol, a type of β2-agonist, promotes hypertrophy in skeletal muscle fibers^21^ by its ability to boost lean mass and reduce fat mass^22,23^. As a result, enhancements in glucose homeostasis could be due to one or both of such effects^24,25^.In aquaculture, tilapia ranks as the second most important finfish species cultivated globally, with Nile tilapia (*Oreochromis niloticus*) being the predominant variety in worldwide production^26^, furthermore, it is estimated that by 2030, it will account for 62% of the total worldwide aquaculture production^27^. Limited studies on Nile tilapia have explored how a high-fat diet (HFD) linked to obesity interacts with the lipid-reducing properties of clenbuterol. Therefore, our study aims to assess the metabolic impacts and the molecular mechanisms of clenbuterol when included in the diet, focusing on growth performance, hematobiochemical variations, histological changes, and the expression of genes associated with glucose and lipid metabolism in Nile tilapia (*Oreochromis niloticus*) fed both standard and high-fat diets.

## Materials and methods

### Ethical approval

The experiment was carried out on Nile Tilapia (*Oreochromis niloticus*), following the standard operating procedures approved by the Institutional Animal Care and Animal Ethics Committee at the Faculty of Aquatic and Fisheries Sciences, Kafrelsheikh University in Egypt (IAACUC-KSU-028-2022).

### Experimental design

The research was conducted at the Department of Fish Processing and Biotechnology, within the Faculty of Aquatic and Fisheries Sciences at Kafrelsheikh University in Egypt. A total of 220 juvenile Nile tilapia with an initial mean weight of 17.25 ± 0.05 g (Mean ± SD) at the beginning of the experimental feeding trial. Fish were reared in a private fish farm in Kafrelsheikh, Egypt. They were acclimatized to the experimental setup for two weeks in glass aquariums, during which they were fed a basal diet twice daily (Table 2). Subsequently, they were evenly distributed across eighteen glass aquariums (80 × 45 × 35 cm, 12 fish per tank) with three replicates for each treatment (six treatments as shown in Table 1). Each aquarium was fitted with a mechanical filter (JAD, China) to remove waste from the water and an air stone to ensure oxygen supply. During the 60-day experiment, water quality parameters such as temperature, dissolved oxygen, and pH were daily monitored. The average recorded values were 26.19 ± 4 °C for temperature, 5.9 ± 0.8 mg/L for dissolved oxygen, and 7.50 ± 0.1 for pH. Additionally, the total ammonia level was checked weekly. All fish groups received feedings twice daily at 8:00 AM and 3:00 PM, with each feeding consisting of 2% of their total body weight.

**Table 1:** Experimental design.

Group code	Treatments
F6Clen0	Normal fat diet (6% fat and 0% clenbuterol)
F6Clen5	Normal fat diet + low clenbuterol (5 mg/kg)
F6Clen10	Normal fat diet + high clenbuterol (10 mg/kg)
F12Clen0	High fat diet 12% (By adding 63 g of soybean oil to 1 kg of normal fat diet)
F12Clen5	High fat diet + low clenbuterol (5 mg/kg)
F12Clen10	High fat diet + high clenbuterol (10 mg/kg)

### Experimental diets

The experimental diets were formulated in two categories: normal fat diets (NF, 6% fat) and high-fat diets (HF, 12% fat), then each category was divided into three groups: Clenbuterol-free diet (devoid of Clenbuterol), low Clenbuterol diet (5 mg/kg diet), and high Clenbuterol diet (10 mg/kg diet), as outlined in Table 1. The pellets were prepared using a Meat Grinding Machine and were sized at 2.33 mm before being air-dried at room temperature.

### Blood and tissue sample collection

After a 24-h fasting period, fish from all groups were counted and weighed. From each aquarium tank, three fish (9 fish /group) were randomly chosen and anesthetized with FA-100 anesthetic (diluted 1:5000, DS Pharma Animal Health Company, Osaka, Japan). Of these, three fish per tank were designated for blood collection and tissue sampling (including liver, kidney, and intestine). Importantly, Tissue samples were subsequently divided into two parts; the first part was preserved in 10% formalin for further histopathological analysis, while the other part was immediately shocked in liquid nitrogen, stored at − 80 °C for total RNA extraction and gene expression analysis. Furthermore, ten fish per tank (totaling 30 fish per group) were chosen randomly for liver weighting and measurement of liver fat, hepatosomatic index (HSI), and intra-peritoneal fat index (IPF), then the whole fish stored at − 20 °C for chemical analysis of body composition (Table 2).

**Table 2:** Ingredient and proximate compositions (g/kg, as-fed) of the normal and high-fat diets supplemented with low and high doses of clenbuterol at both levels of dietary fat.

	F6Clen0	F6Clen5	F6Clen10	F12Clen0	F12Clen5	F12Clen10
Ingredients (g/kg)						
Fish meal (65%)	100	100	100	100	100	100
Soybean meal (45%)	463	463	463	463	463	463
Corn gluten meal	30	30	30	30	30	30
Yellow corn	164	165	170	135	125	115
Wheat flour	186	180	170	152	157	162
Soybean oil	35	35	35	98	98	98
Vitamin mixture*	8	8	8	8	8	8
Mineral mixture**	5	5	5	5	5	5
DiCaP	6	6	6	6	6	6
Choline chloride	2	2	2	2	2	2
Stay C***	1	1	1	1	1	1
Clenbuterol (100%)	0	5	10	0	5	10
Composition (%)						
Crude protein	32.20	32.14	32.07	31.55	31.53	31.51
DE (Kcal/Kg)	3004.63	2998.17	2997.59	3365.90	3343.24	3320.58
Crude lipid	5.84	5.83	5.84	11.99	11.96	11.94
Ash	5.167	5.166	5.168	5.116	5.10	5.09
Crude fiber	4.11	4.10	4.10	4.00	3.98	3.97
Ca	0.798	0.798	0.797	0.796	0.796	0.796
P	0.824	0.823	0.821	0.804	0.803	0.802

*Vitamin (g/kg premix): Thiamin HCl, 0.44; Riboflavin, 0.63; Pyridoxine HCl, 0.91; DL pantothenic acid, 1.72; Nicotinic acid, 4.58; Biotin, 0.21; Folic acid, 0.55; Inositol, 21.05; Menadione sodium bisulfite, 0.89; Vitamin A acetate, 0.68; Vitamin D3, 0.12; dL-alpha-tocoperol acetate, 12.63; Alpha-cellulose, 955.59.

**Trace mineral (g/100 g premix): Cobalt chloride, 0.004; Cupric sulfate pentahydrate, 0.25; Furrous sulfate, 4.000; Mag-nesium sulfate anhydrous, 13.862; Manganous sulfate monohy-drate, 0.650; Potassium iodide, 0.067; Sodium selenite, 0.010; Zinc sulfate hepahydrate, 13.193; Alpha-cellulose, 67.964.

***Stay C®, (L-ascorbyl-2-polyphosphate 35%).

### Hematological and serum biochemical analysis

At the conclusion of the experiment, two blood samples were collected from the caudal vein using a 3 mL disposable syringe. The first blood sample an anticoagulant; EDTA (Ethylene diamine tetra acetic acid) to quantify erythrocytes, hemoglobin (Hb), packed cell volume (PCV), total leukocyte counts and differential leucocyte count (monocytes, lymphocytes, heterophils, eosinophils,) according to^28^. The second blood sample was collected without anticoagulant, centrifuged at 3000 rpm at 4 °C for 10 min using a bench-top undercooling centrifuge (Heraeus Megafuge 8R, Thermo Fisher Scientific, Germany) for separation of the serum, then stored at − 20 °C until biochemical analyses. Commercial kits (BIODIGNOSTIC, Giza, Egypt) were used to test serum for total proteins, albumin, cholesterol, triglycerides, high-density lipoprotein-C (HDL-C), glucose, urea, creatinine, alanine aminotransferase (ALT), aspartate aminotransferase (AST), and lactate dehydrogenase (LDH). Globulins concentration (Glob) was determined by subtracting albumin values from total, and consequently albumin to globulin ratio (A/G) was estimated. VLDL-C concentrations were determined using Friedewald standard equation^29^.

### Histopathology study

Collected fish were euthanized by using MS222 at a concentration of 25 mg/L of water. Liver, kidney, and intestinal specimens were soaked in 10% phosphate-buffered neutral formalin (dehydrated and cleaned in xylene), then processed into paraffin blocks and sliced off at 5 μm thickness. Standard histological techniques were used to stain the sections, including hematoxylin and eosin. Images were taken with an inverted light microscope at the Institute of Nanoscience and Nanotechnology, Kafrelsheikh University, Egypt (Leica Microsystems-Fluorescence Model, DMi8 manual, Germany).

### Total RNA extraction, cDNA synthesis, and rt-qPCR assay

Total RNA was extracted from 50 mg of the livers and muscle of *Oreochromis niloticus* using GENEzol ™ Reagent (Gene aid, UK) following the manufacturer’s directions. RNA integrity was validated using ethidium bromide-stained 2% agarose gel electrophoresis. The concentration as well as purity of RNA samples were measured using a Nanodrop BioDrop spectrophotometer (Biochrom Ltd, Cambridge CB23 6DW, UK) based on the A260/A280 nm ratio. Five μg of RNA samples were reverse transcribed using the TOP script TM RT Dry Mix (dt18/dN6 plus) kit (enzynomics, Daejeon, South Korea). Gene expression analysis was performed in Rotor Gene-Q (Qiagen-Germany) with Tilapia-specific primers for the amplification of Immune-related genes as *tumor necrosis factor alpha* (*tnfa*), *interleukin-1β* (*il1b*). Antioxidant genes as *nuclear factor -E2-related factor 2 (nrf2), kelch-like ECH-associated protein 1 (keap1)*, *glutathione peroxidase* (*gp*x), *superoxide dismutase* (*sod2*) (Table 3)^30–35^. The amplification reaction was done using TOPrealTM qPCR 2X PreMIX (SYBR Green with Low Rox) kit (enzynomics, Daejeon, South Korea). The reaction volume of 20 μl mix of 10 µL SYBR Green, 0.6 µL of both forward and reverse primer, 1 µL of template cDNA, and the total volume of 20 µL adjusted with nuclease-free water. The analysis of melt curve was performed to ensure the specificity of amplification at 72 °C to 95 °C. The qPCR data was normalized using the geometric averaging of two internal reference genes; 18S ribosomal RNA (*18 s rRNA*) and Ubiquitin C (*ubce*) to calculate fold change). All genes were examined in triplicate. CT values for every sample were determined and included in Efficiency-corrected fold change (2^−ΔΔCT^) calculation based on the Livak and Schmittgen^36^.

**Table 3:** Primers used for qRT-PCR analysis.

Gene	Gene name	Primer sequence (5′–3′)	NCBI gene bank Accession No	References
Internal reference genes				
*18s rRNA* F	18 s ribosomal rRNA	GGACACGGAAAGGATTGACAG	JF698683	^30^
*18 s rRNA* R		TTCGTTATCGGAATTAACCAGA		
*ubce* F	Ubiquitin C	CTCTCAAATCAATGCCACTTCC	XM_003460024	
*ubce* R		CCCTGGTGGAGGTTCCTTGT		
Anti-oxidant genes				
*nrf2* F	Nuclear factor -E2-related factor 2	CTGCCGTAAACGCAAGATGG	XM_003447296.4	^31^
*nrf2* R		ATCCGTTGACTGCTGAAGGG		
*keap1* F	Kelch-like ECH-associated protein 1	CTTCGCCATCATGAACGAGC	XM_003447926.3	
*keap1* R		CACCAACTCCATACCGCACT		
*sod2* F	Superoxide dismutase	CATGCCTTCGGAGACAACAC	AY491056.1	
*sod2* R		ACCTTCTCGTGGATCACCAT		
Immune-related genes				
*il1b* F	Interleukin 1, beta	CAAGGATGACGACAAGCCAACC	XM_003460625.2	^32^
*il1b* R		AGCGGACAGACATGAGAGTGC		
*tnfa* F	Tumor necrosis factor a	GGAAGCAGCTCCACTCTGATGA	JF957373.1	^33^
*tnfa* R		CACAGCGTGTCTCCTTCGTTCA		
*il10* F	Interleukin-10	GCAGAACCGTGTCCAGGTAA	XM_003441366.2	^32^
*il10* R		CTGCTAGATCAGTCCGTCGAA		
Fat metabolism related genes				
*cpt1* F	Carnitine palmitoyltransferase 1	TTTCCAGGCCTCCTTACCCA	XM_013268638.3	^34^
*cpt1* R		TTGTACTGCTCATTGTCCAGCAGA		
*ppara F*	Peroxisome proliferator-activated receptor alpha	CTGATAAAGCTTCGGGCTTCCA	KF871430.1	
*ppara R*		GCTCACACTTATCATACTCCAGCT		
*acox1* F	Acyl-coenzyme A oxidase 1	AGTCCCACTGTGAGCTCCATCAA	XM_003447910.5	
*acox1* R		CAGACCATGGCAGTTTCCAAGA		
*dgat2* F	Diacylglycerol O-acyltransferase 2	GCTTGAATTCTGTCACCCTGAAGA	XM_003458972.4	^34^
*dgat2* R		ACCTGCTTGTAGGCGTCGTTCT		
*srebp* F	Sterol regulatory element binding protein	TGCAGCAGAGAGACTGTATCCGA	XM_005457771.3	
*srebp R*		ACTGCCCTGAATGTGTTCAGACA		
*fas* F	Fatty acid synthetase	TGAAACTGAAGCCTTGTGTGCC	GU433188.1	^35^
*fas* R		TCCCTGTGAGCGGAGGTGATTA		
*lpl* F	Lipoprotein lipase a	TGCTAATGTGATTGTGGTGGAC	GU433189.1	
*lpl* R		GCTGATTTTGTGGTTGGTAAGG		
*cd36* F	Cluster of differentiation 36	ATCTTCGAACCATCCATGTCAGT	XM_003452029.2	^34^
*cd36* R		GATATGTGATGCTGGAGGAAGCAA		
Glucose metabolism related genes				
*g6pase* F	Glucose-6-phosphatase	AGCGCGAGCCTGAAGAAGTACT	XM_003448671	^35^
*g6pase* R		ATGGTCCACAGCAGGTCCACAT		
*pepck* F	Phosphoenolpyruvate carboxy kinase	GCCCTCAGTCCAGCTGTAAG	XM_003448375.4	
*pepck* R		CACATCCCTCGGGTCAGTTC		
*pk* F	Pyruvate d kinase	CCGTAAGGCTGCAGACGTGCA	DQ066876.1	
*pk* R		ATCTGCGCACGCCCTCATGG		

The listed Primers Sequences, accession number of the targeted gene, references. (F) the forward sequence of the tested primers, (R) the reverse sequence of the tested primers.

### Statistical analysis

Data are demonstrated as mean ± standard error of the mean (M ± SEM). Preliminary, Shapiro–Wilk’s test was employed to assess data normality, while Levene’s test was used to evaluate variance homogeneity, both conducted with a significance level set at *p* < 0.05. Percentage data were subjected to Arcsine transformation prior to Analysis of variances. To investigate the differential effects of high fat, Clenbuterol, and their interaction, body indices, growth performances, hematological parameters, biochemical analyses, histomorphometric measurements, and relative gene expression were analyzed using Two-way ANOVA, pursued by Tukey’s multiple comparison test (*p* < 0.05). All statistical analyses ware conducted using GraphPad Prism (version 9.5, GraphPad Software, San Diego, California, USA).

## Results

### Growth performance indices, feed utilization, survival, and body nutritional composition

As shown in Table 4, clenbuterol at the chosen doses showed nonsignificant change in FBW, BWG, feed intake, SGR, FCR, PER compared with the normal control group. Meanwhile, HFD reduced FBW, BWG, and PER while raised FCR, IPF, liver fat %, and body ash %, but reduced body protein% and fat %. Furthermore, clenbuterol dietary inclusion to HFD markedly increased FBW, BWG, PER while reduced HSI, IPF ,liver fat %, body fat % with raised body protein % and body ash % when matched to the HFD treatment (*P* < 0·05) (Table 4).

**Table 4:** Growth and feed utilization parameters of Nile tilapia (*Oreochromis niloticus*) fed on normal and high fat diets supplemented with low and high dose of clenbuterol at both level of dietary fat for 8 weeks.

Parameters	F6Clen0	F6CLen5	F6Clen10	F12Clen0	F12Clen5	F12Clen10	*P* value of two-way ANOVA		
							Fat	Clenbuterol	Interaction
IBW (g)	17.42 ± 0.63	17.25 ± 0.16	17.25 ± 0.05	17.13 ± 0.03	17.21 ± .07	17.29 ± 0.18			
FBW (g)	35.80 ± 0.80^ab^	34.45 ± 0.67^ac^	33.49 ± 047^bc^	32.29 ± 0.70^c^	36.03 ± 0.32^ab^	36.39 ± 0.72^a^	0.5397	0.1701	< 0.0001
BWG (g)	18.38 ± 0.94^a^	17.20 ± 0.52^ab^	16.24 ± 0.24^ab^	15.17 ± 0.85^b^	18.83 ± 0.72^a^	19.10 ± .53^a^	0.4521	0.1947	0.0004
Total feed intake(g)	28.20 ± 0.09^a^	36.10 ± 0.54^ab^	35.50 ± 0.09^ab^	36.40 ± 1.06^ab^	38.10 ± 0.67^a^	38.10 ± 0.40^a^	0.0623	0.6941	0.0018
SGR (% day-1)	1.11 ± 0.05	1.06 ± 0.04	1.02 ± 0.04	0.97 ± 0.09	1.13 ± 0.23	1.14 ± 0.03	0.999	0.632	0.2774
FCR	2.08 ± 0.09	2.10 ± 0.07	2.19 ± 0.05	2.41 ± 0.14	2.09 ± 0.43	2.00 ± 0.08	0.741	0.5604	0.281
PER	1.60 ± 0.07^a^	1.58 ± 0.03^a^	1.52 ± 0.01^a^	1.33 ± 0.05^b^	1.63 ± 0.01^a^	1.67 ± 0.03^a^	0.4585	0.0016	< 0.0001
Survival (%)	95.83 ± 5.9	100 ± 2.36	100 ± 2.63	91.67 ± 5.3	97.17 ± 5.2	93.33 ± 5.3	0.0409	0.2483	0.7260
HSI (%)	2.85 ± 0.14^ab^	2.91 ± 0.12^a^	2.19 ± 0.20^b^	3 ± 0.17^a^	3.26 ± 0.1^a^	2.63 ± 0.13^ab^	0.0180	0.0005	0.6132
IPF (%)	0.53 ± 0.05^c^	0.3 ± 0.05^cd^	0.08 ± 0.04^d^	1.19 ± 0.06^a^	0.93 ± 0.07^b^	0.31 ± 0.03^cd^	< 0.0001	< 0.0001	0.0011
Liver fat (%)	14.29 ± 0.62^b^	11.67 ± 0.60^bc^	3.3 ± 0.65^d^	20 ± 0.85^a^	9.46 ± 0.5^c^	10 ± 0.62^e^	< 0.0001	< 0.0001	< 0.0001
Body fat (%)	29.75 ± 0.36^a^	22.93 ± 0.47^d^	27.47 ± 0.46^bc^	28.28 ± 0.40^ab^	29.54 ± 0.38^a^	26.06 ± 0.50^c^	0.0018	< 0.0001	< 0.0001
Body protein (%)	52 ± 0.58^b^	54.82 ± 0.54^a^	54.84 ± 0.62^a^	48.44 ± 0.49^c^	52.11 ± 0.72^b^	52.44 ± 0.75^ab^	< 0.0001	< 0.0001	0.6341
Body ash (%)	10.48 ± 0.43^d^	14.29 ± 0.59^bc^	14.68 ± 0.54^bc^	13.50 ± 0.49^c^	16.08 ± 0.72^ab^	17.99 ± 0.62^a^	< 0.0001	< 0.0001	0.3846

Growth and feed utilization parameters of Nile tilapia (*Oreochromis niloticus*) fed on normal and high fat diets supplemented with low and high dose of clenbuterol at both level of dietary fat. IBW; Initial body weight, FBW; final body weight, BWG; body weight gain, SGR; specific growth rate, FCR; Feed conversion ratio, PER; protein efficiency rate, HSI; hepatosomatic index, IPF; intraperitoneal fat index. Data are expressed as Mean ± SEM where n = 3 as triplicate tanks for BWG%, FCR, SGR, PER, HSI, IPF, liver at, body fat, body protein and body ash and n = 45 for IBW and FBW. Values with different superscripts within a row are significantly different (*p* < 0.05).

### Hematological parameters

Clenbuterol supplementation at the chosen doses revealed a significant increase in RBCs and Hb While showing non-significant increase in PCV, WBCs and white blood cell differential counts compared with the normal fat-control fed group (Table 5). Moreover, a High-fat diet showed a significant reduction in PCV and Hb concentration exhibiting microcytic hypochromic anemia while increased WBCs, lymphocytes and monocytic count compared with the normal fat control group. Interestingly, clenbuterol dietary incorporation raised RBCs count, PCV and HB concentrations and restored the WBCs, heterophils, lymphocytes and monocytes to normal compared with the high fat diet fed group with the best response to the high dose of clenbuterol.

**Table 5:** Hematological parameters of Nile tilapia (*Oreochromis niloticus*) fed on normal and high fat diets supplemented with low and high dose of clenbuterol at both level of dietary fat for 8 weeks.

Parameters	F6Clen0	F6CLen5	F6Clen10	F12Clen0	F12Clen5	F12Clen10	*P* value of two-way ANOVA		
							Fat	Clenbuterol	Interaction
RBCs									
(× 10^6^/µL)	2.87 ± 0.03^b^	3.09 ± 0.05^a^	3.22 ± 0.1^a^	2.8 ± 0.03^b^	2.54^1^ ± 0.3^c^	3.24 ± 0.06^a^	0.0217	0.0019	0.1248
Hb (g/dL)	8.71 ± 0.04^cc^	9.47 ± 0.13^b^	9.88 ± 0.08^a^	8.00 ± 0.08^d^	8.43 ± 0.08^cd^	9.57 ± 0.1^ab^	< 0.0001	< 0.0001	0.0013
PCV (%)	28 ± 0.63^a^	30.33 ± 0.68^a^	31.33 ± 1.12^a^	26.67 ± 2.62^b^	24.66 ± 0.73^b^	31.66 ± 0.68^a^	0.0115	0.0014	0.0848
MCV (fL)	97.56 ± 1.02^a^	98.16 ± 0.65^a^	97.29 ± 0.41	95.25 ± 1.51^b^	97.09 ± 3.50^a^	97.72 ± 7.17^a^	0.0416	0.0176	0.9329
MCH (pg)	30.35 ± 0.21^a^	30.65 ± 0.22^a^	30.68 ± 0.13^a^	28.57 ± 0.65^b^	33.19 ± 0.29^a^	29.54 ± 0.23^b^	0.0160	< 0.0560	< 0.0360
MCHC (g/dL)	31.11 ± 0.54^a^	31.22 ± 0.02^a^	31.54 ± 0.21^a^	29.9 ± .042^b^	34.18 ± 0.32^a^	30.23 ± 0.35^a^	0.0142	0.0346	0.0244
WBCs (× 10^3^/μL)	20.80 ± 0.41^b^	22.01 ± 0.68^ab^	21.36 ± 0.3^ab^	24.80 ± 1.39^a^	19.45 ± 0.4^b^	22.61 ± 1.36^ab^	0.2245	0.0815	0.0040
Heterophils (× 10^3^/μL)	3.22 ± 0.001^a^	2.55 ± 0.13^ab^	2.2 ± 0.06^b^	3.15 ± 0.22^a^	2.3 ± 0.22^b^	2.59 ± 0.31^ab^	0.8821	0.0003	0.2324
Lymphocytes (× 10^3^/μL)	15.71 ± 0.51^b^	17.27 ± 0.77^ab^	17.39 ± 0.79^ab^	19.32 ± 0.98^a^	15.08 ± 0.49^b^	17.91 ± 0.96^ab^	0.3118	0.1295	0.0039
Monocytes (× 10^3^/μL)	1.46 ± 0.03^bc^	1.83 ± 0.03^ab^	1.49 ± 0.08^ac^	1.90 ± 0.16^a^	1.35 ± 0.04^c^	1.74 ± 0.15^ab^	0.3468	0.6201	0.0002
Eosinophils (× 10^3^/μL)	0.21 ± 0.003	0.22 ± 0.01	0.15 ± 0.02	0.17 ± 0.07	0.23 ± 0.02	0.23 ± 0.004	0.4973	0.3539	0.1444
Basophils (× 10^3^/μL)	0.20 ± 0.13	0.15 ± 0.01	0.14 ± 0.01	0.25 ± 0.01	0.22 ± 0.01	0.14 ± 0.01	0.3683	0.3001	0.7571

Hematological parameters of Nile tilapia (*Oreochromis niloticus*) fed on normal and high fat diets supplemented with low and high dose of clenbuterol at both level of dietary fat for 8 weeks. RBCs; red blood corpuscles, Hb; Hemoglobin, PCV; packed cell volume, MCV; Mean Cell Volume, MCH; Mean Cell Hemoglobin. WBCs; White blood cells. Data are expressed as Mean ± SEM where n = 5. For each parameter, values with different superscripts are significantly different (*p* < 0.05). Small letters indicate significant differences (two-way ANOVA).

### Serum biochemical profiles

The results revealed that dietary supplementation with clenbuterol did not change the normal liver synthetic function as evidenced by the non-significant alterations in serum albumin concentration (Table 6). On top of that, total proteins and globulins concentrations were shown to be significantly elevated in a dose-dependent manner matched to the control group. Clenbuterol treatment revealed a significant increase in serum AST, ALT and LDH activities compared to the control group. Moreover, HFD revealed a marked elevation of serum ALT, AST, and LDH compared with the normal control group. Clenbuterol ameliorated the elevated activities of ALT, AST, and LDH compared with the HFD-fed group.

**Table 6:** Biochemical parameters of Nile tilapia (*Oreochromis niloticus*) reared for 8 weeks and fed on normal and high fat diets supplemented with low and high dose of clenbuterol at both level of dietary fat.

Parameters	F6Clen0	F6CLen5	F6Clen10	F12Clen0	F12Clen5	F12Clen10	*p* value of two-way ANOVA		
							Fat	Clenbuterol	Interaction
Total protein (g/dL)	4.90 ± 0.08^bc^	5.63 ± .28^b^	6.83 ± 0.09^a^	3.77 ± 0.23^d^	4.80 ± 0.32^bc^	4.30 ± 0.27^cd^	< 0.0001	< 0.0001	0.0027
Albumin (g/dL)	1.24 ± 0.02^a^	1.20 ± 0.03^a^	1.25 ± 0.06^a^	0.73 ± 0.18^b^	1.15 ± 0.08^a^	1 ± 0.07^ab^	0.0014	0.1107	0.0645
Globulins (g/dL)	3.66 ± 0.1^b^	4.43 ± 0.25^ab^	5.8 ± 0.44^a^	3.03 ± 0.05^b^	3.65 ± 0.25^b^	3.3 ± 0.20^b^	0.0015	0.0498	0.1162
A/G	0.34 ± 0.015	0.27 ± .001	0.25 ± .05	0.24 ± 1.65	0.32 ± 0.01	0.30 ± 0.01	0.5436	0.4131	0.0169
HDL-C (mg/dL)	48.33 ± 2.58^a^	50.67 ± 1.57^ac^	53.67 ± 2.84^bc^	31.33 ± 0.54^c^	45 ± 2.37^ab^	38 ± 2.45^ac^	0.2456	0.0677	0.0010
VLDL-C (mg/dL)	28.80 ± 1.41^b^	30.73 ± 1.75^b^	28.80 ± 0.94^b^	55.53 ± 1.63^a^	31.27 ± 1.62^b^	28.87 ± 3.07^b^	0.0080	0.0086	0.0034
LDL-C (mg/dL)	97.87 ± 9.73^bc^	100.60 ± 10.56^bc^	91.53 ± 15.82^bc^	190.81 ± 7.47^a^	127.40 ± 8.07^b^	63.47 ± 11.41^c^	0.0120	< 0.0001	< 0.0001
TC (mg/dL)	175 ± 8.50^bc^	182 ± 18.44^bc^	174 ± 14.78^bc^	277.67 ± 9.64^a^	203.67 ± 17.28^b^	130.33 ± 11.19^c^	0.0123	< 0.0001	< 0.0001
TG (mg/dL)	144 ± 7.03^b^	153.67 ± 13.21^ab^	144 ± 15.90^b^	277.67 ± 8.13^a^	156.33 ± 14.79^ab^	144.33 ± 15.36^b^	0.0485	0.0681	0.0381
Glucose (mg/dL)	42.33 ± 2.73^b^	44 ± 0.45^b^	43 ± 0.45^b^	65.67 ± 2.91^a^	48 ± 2.68^b^	44.67 ± 0.68^b^	< 0.0001	0.0006	0.0002
Creatinine (mg/dL)	0.45 ± 0.004^b^	0.52 ± 0.04^b^	0.42 ± 0.04^b^	0.96 ± 0.02^a^	0.51 ± 0.23^b^	0.45 ± 0.15^b^	0.0182	0.0115	0.0100
BUN (mg/dL)	8 ± 1.61^ab^	7.67 ± 0.24^ab^	5.67 ± 0.26^b^	9.67 ± 0.24^a^	6.67 ± 1.1^ab^	6.30 ± 0.22^b^	0.2441	0.1555	0.8021
Ast (U/L)	95.13 ± 0.46^d^	135.17 ± 2.12^c^	150.83 ± 2.45^b^	196.67 ± 4.64^a^	142.33 ± 3.04^b^	138 ± 3.82^bc^	< 0.0001	< 0.0001	0.1608
Alt (U/L)	30 ± 0.99^c^	44 ± 3.49^c^	55 ± 4.47^b^	85.67 ± 2.70^a^	70 ± 1.25^b^	53.33 ± 1.19^b^	< 0.0001	< 0.0001	0.0638
Ldh (U/L)	268.50 ± 4.74^c^	287.33 ± 6.35^b^	294 ± 3.58^b^	394.33 ± 7.38^a^	302.67 ± 2.27^b^	281.33 ± 5.80^bc^	< 0.0001	< 0.0001	< 0.0001

Biochemical parameters of Nile tilapia (*Oreochromis niloticus*) reared for 8 weeks and fed on normal and high fat diets supplemented with low and high dose of clenbuterol at both level of dietary fat. for total protein, cholesterol, triglycerides, high density lipoprotein (HDL), very low-density lipoprotein (VLDL), low density lipoprotein (LDL), Urea, creatinine, Glucose, albumin, globulin, albumin/globulin (A/G) ratio, aspartate aminotransferase (Ast), alanine transaminase (Alt) and lactate dehydrogenases (Ldh). Data are expressed as Mean ± SEM where n = 5. small letters indicate significant differences (two-way ANOVA).

However, HDF showed reduced total proteins, albumin, globulins and HDL-C concentrations while raised glucose and lipid profile components including TC, TG, VLDL-C, and LDL-C levels together with elevated serum hepatic injury biomarkers such as ALT, AST and LDH enzymes, and raised kidney injury biomarkers such as BUN and creatinine compared with the control group. Interestingly, clenbuterol dietary incorporation to HFD-fed fish succeeded in modulating the aforementioned findings to their normal levels.

### Hepatic, renal, and intestinal histological analysis

**Fig. 1:**
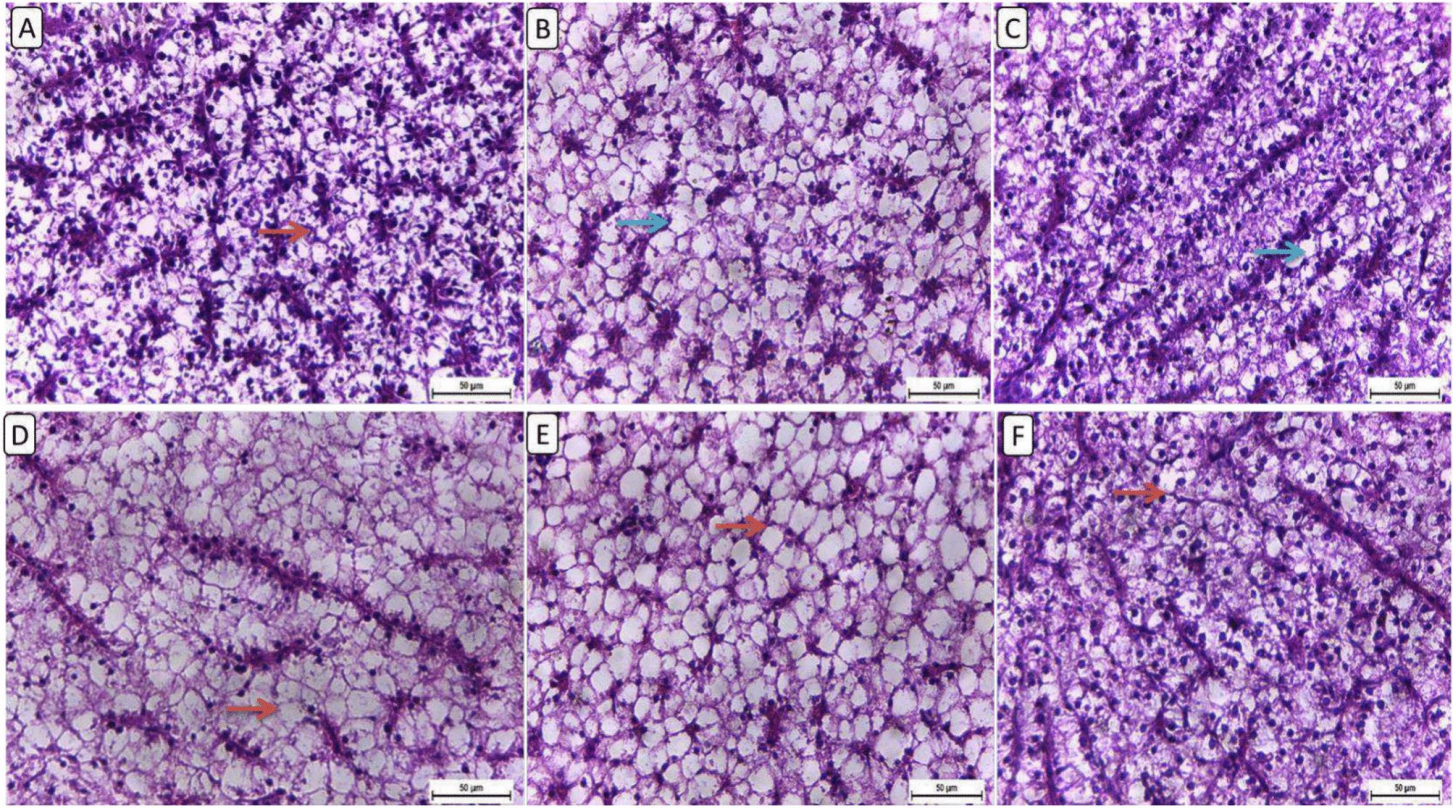
Representative photomicrograph of liver from different treatment groups. (**A**) NF6% showing mild to moderate hepatic vacuolation. (**B**) NF + Low clenbuterol showing diffuse hepatic vacuolation (thin arrow). (**C**) NF + High clenbuterol showing mild hepatic vacuolation (thin arrow). (**D**) HF group showing diffuse, severe hepatic vacuolation with clear, difinite intracytoplacmic vacuoles (thin arrow). (**E**) HF + Low clenbuterol group showing diffuse marked hapatic vacuolation with intracytoplasmic clear, fat vacuoles pushing nucleus peripherally (thin arrow). (**F**) HF + High clenbuterol showing mild hepatic vacuolation (thin arrow). Scale bar = 50. (Figure A and D for the control group after Rashwan et al., 2024).

Histological analysis of liver sections from various groups (Fig. 1), the control group exhibited mostly normal characteristics, including normal lobular architecture and hepatocytes with mild to moderate hepatic vacuolation (Fig. 1A). Hepatocytes in the F6 clenbu5 group seemed normal, showing diffuse hepatic vacuolation (Fig. 1B). In response to clenbuterol’s high dose, there was a diffuse, marked hepatic vacuolation (Fig. 1C). In contrast, the HFD group had prominent widespread hepatic vacuolation with distinct intra-cytoplasmic vacuoles (Fig. 1D). The F12 Clenb 5 group had diffuse hepatic vacuolation with intracytoplasmic fat vacuoles pushing the nucleus peripherally (Fig. 1E). While the liver sections from the F12clenb 10 group showed mild hepatic vacuolation (Fig. 1F).

**Fig. 2:**
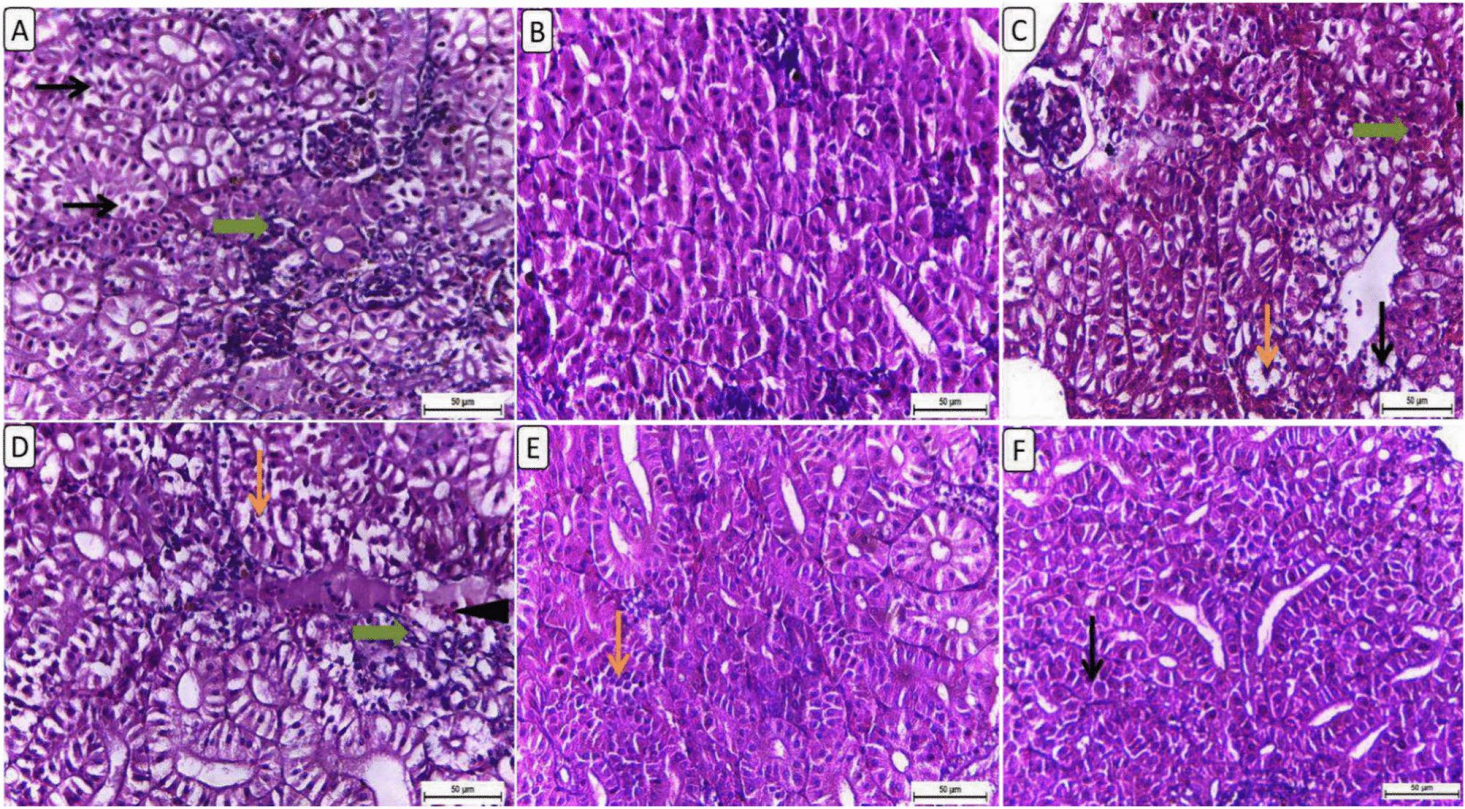
Representative photomicrograph of kidney from different treatment groups. (**A**) NF6% showing moderate tubular necrosis (thin arrows) and desquamation (thin arrows) with glomerular shrunken (thick arrow). (**B**) NF + Low clenbuterol showing normal histological appearance of renal tubules. (**C**) NF + High clenbuterol showing focal tubular vacuolation (thin arrows) with few tubular necrosis and minimal interstitial hemorrhage (thick arrow). (**D**) HF group showing diffuse tubular damage represented by severe tubular vacuolation and necrosis (thin arrows), interstitial fibrosis (thick arrow) admixed with lymphocytes and numerous RBCs (arrowhead). (**E**) HF + Low clenbuterol group showing normal histological appearnce of most tubules except, few, occasional individualized tubular epithelial cells (thin arrow). (**F**) HF + High clenbuterol showing normal histological appearance of most renal tubules with few, occasional necrotic cells (thin arrow). Scale bar = 50 μm. (Figure A and D for the control group after Rashwan et al., 2024).

Figure 2 showed a histological study of kidney sections from different treatment groups, which revealed significant differences in renal architecture and cellular morphology. The kidney in the control group had a histologically normal tubular and glomerular structure (Fig. 2A). The F6Clenb5 group exhibited a normal histological appearance of renal tubules (Fig. 2B). The kidney sections from the F6Clenb10 group showed focal tubular vacuolation with little tubular necrosis and minimal interstitial hemorrhage (Fig. 2C). The F12Clenb0 group exhibited diffuse tubular damage represented by severe tubular vacuolation and necrosis with interstitial fibrosis with mixed lymphocytes and numerous RBCs (Fig. 2D). The kidney in the F12Clenb5 group showed a normal histological appearance of most tubules except, few, occasional individualized tubular epithelial cells (Fig. 2E). Furthermore, the kidney sections from the F12Clenb10 group displayed normal histological appearance of most renal tubules with few, occasional necrotic cells (Fig. 2F).

**Fig. 3:**
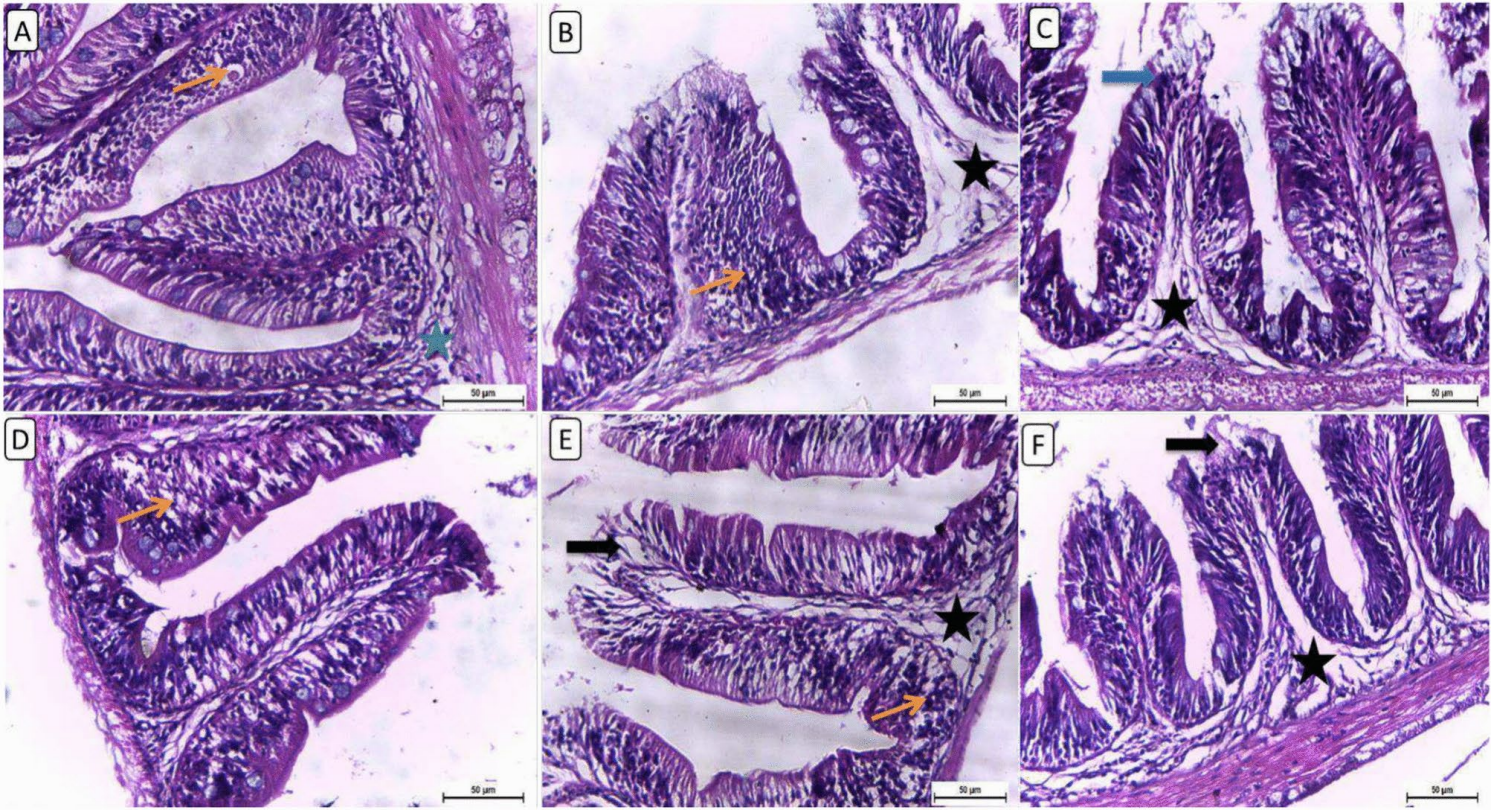
Representative photomicrograph of intestine from different treatment groups. (**A**) NF6% showing NF6% showing occasional apoptotic bodies (thin arrow), numerous goblet cells, few submucosal edema with few inflammatory cells (star). (**B**) NF + Low clenbuterol showing thickened, shortened intestinal villi expanded with severe lamina proprial edema (star) and lymphocytic infiltrations (thin arrow). (**C**) NF + High clenbuterol showing few apical mucosal loss (thick arrow) with moderate submucosal edema (star) leading to moderate expanded intestinal mucosa. (**D**) HF group showing mild to moderate expanded, shortened intestinal villi with many vacuoles (thin arrow). (**E**) HF + Low clenbuterol group showing mild intestinal thickening with vacuolation (thick arrow), mild lymphocytic infiltration (thin arrow) and mild lamina proprial edema (star). (**F**) HF + High clenbuterol showing apical mucosal loss (thick arrow), diffuse submucosal edema admixed with inflammatory cells (star). Scale bar = 50 μm. (Figure A and D for the control group after Rashwan et al., 2024).

Histopathological analysis of intestinal sections from different treatment groups revealed varied findings in intestinal architecture and mucosal integrity (Fig. 3). The intestine in the control group has occasional apoptotic bodies, numerous goblet cells, and few submucosal edema with few inflammatory cells (Fig. 3A). F6Clenb5 showed thickened, shortened intestinal villi expanded with severe edema in lamina propria with lymphocytic infiltrations (Fig. 3B). F6Clenb10 showed few apical mucosal loss with moderate submucosal edema leading to moderately expanded intestinal mucosa. The F12Clenb0 group, revealed mild to moderate expanded, shortened intestinal villi with many vacuoles (Fig. 3D). F12Clenb5 group, showed mild intestinal thickening with vacuolation, mild lymphocytic infiltration and mild oedema in lamina propria (Fig. 3E). Furthermore, the F12Clenb10 group had an apical mucosal loss, diffuse submucosal edema and mixed with inflammatory cells (Fig. 3F).

### Histo-morphometric of muscle fibers

As explained in Table 7, in comparison with the control group, clenbuterol clenbuterol-supplemented group revealed increased muscle fiber count, total area, and area % in a dose–response manner. Moreover, HFD and clenbuterol-supplemented groups to HFD showed non-significant change in muscle fiber count, total area, average size, and area % compared with the normal control group.

**Table 7:** Histomorphometric of muscle fibers of Nile tilapia (*Oreochromis niloticus*) reared for 8 weeks and fed on normal and high fat diets supplemented with low and high dose of clenbuterol at both level of dietary fat.

Parameters	F6Clen0	F6CLen5	F6Clen10	F12Clen0	F12Clen5	F12Clen10	*p* value of two-way ANOVA		
							Fat	Clenbuterol	Interaction
Count (n)	567 ± 25.38^b^	767.78 ± 11.85^a^	803.56 ± 9.04^a^	537.89 ± 28.12^b^	548.44 ± 38.58^b^	577.67 ± 37.07^b^	< 0.0001	0.0001	0.0021
Total area (µm^2^)	16,742.24 ± 794.06^b^	22,344.73 ± 443.08^a^	23,400.22 ± 299.87^a^	15,748.05 ± 1221.6^b^	16,715.73 ± 1580.90^b^	16,448.15 ± 930.45^b^	0.0019	< 0.0001	0.0161
Average size(µm^2^)	29.50 ± 0.32	29.09 ± 0.25	29.12 ± 0.21	29.1 ± 0.82	30.30 ± 1.17	28.55 ± 0.52	0.4623	0.9000	0.3702
Area (%)	25.79 ± 0.54^b^	30.37 ± 0.29^a^	31.12 ± 0.27^a^	24.92 ± 0.76^b^	25.73 ± 1.06^b^	25.15 ± 0.31^b^	< 0.0001	0.0002	0.0012

### Differential gene expression analysis

#### Antioxidative function-related gene expression

**Fig. 4:**
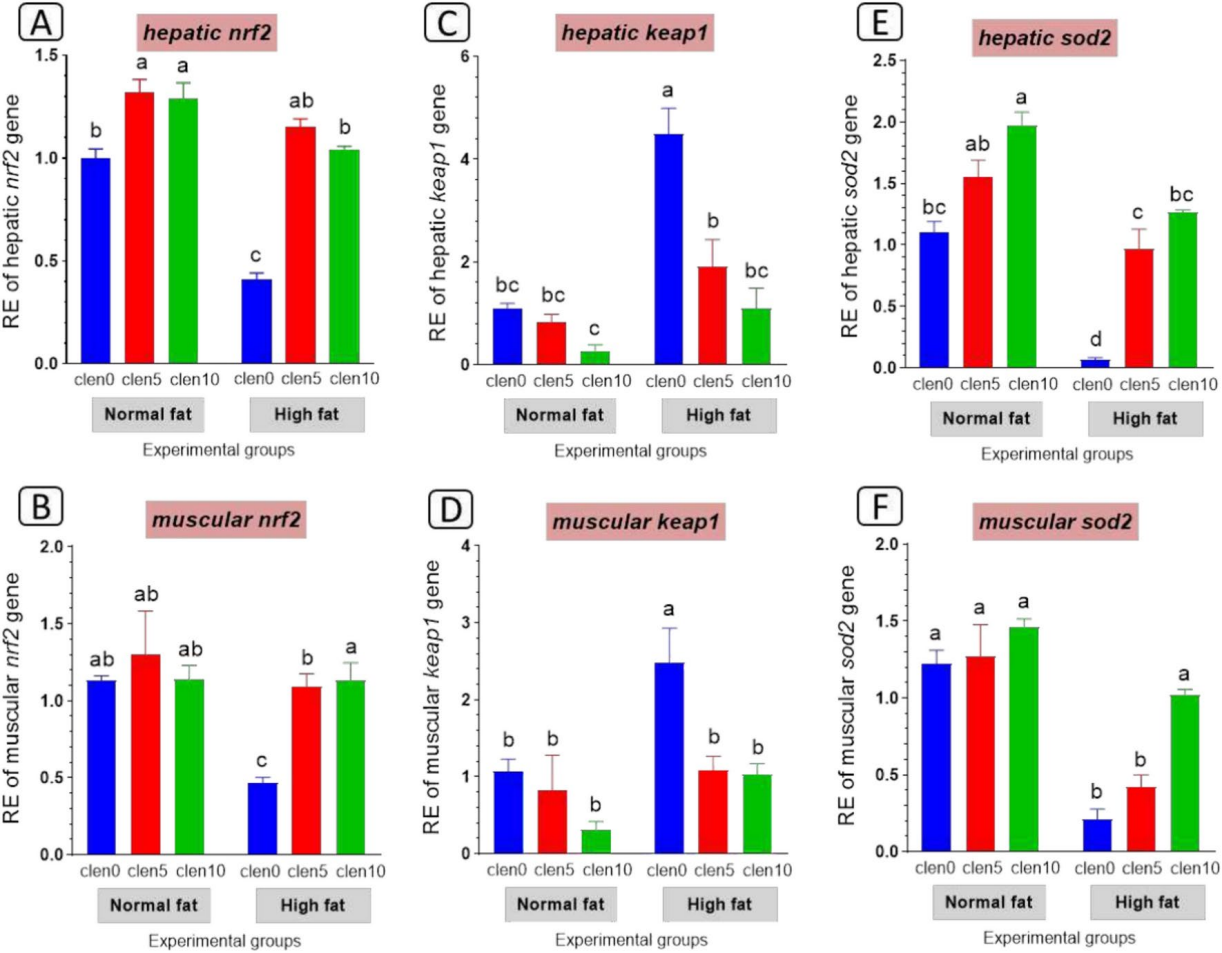
Expression of different anti-oxidant genes in liver and muscle of Nile tilapia groups fed on normal and high fat diets with clenbuterol. (**A** and **B**) Nuclear factor erythroid 2-related factor 2: *nrf 2*, (**C** and **D**) Kelch-like ECH-associated protein 1: *keap 1*, (**E** and **F**) superoxide dismutase2: *sod2*. *P* value results of the two-way ANOVA are represented on the top of each figure. Columns with different superscript letters in the same figure are significantly different (*p* ≤ 0.05).

Antioxidant status was evident in the liver and muscle as portrayed in Fig. 4. HFD administration resulted in reduced hepatic and muscular expression levels of *nrf2* (Fig. 4A), and *sod* (Fig. 4C) while raised *Keap1* (Fig. 4B) compared with the normal fat-fed fish group. On the other hand clenbuterol supplementation activates *nrf2* signaling via enhanced hepatic and muscular expression levels of *nrf2* (Fig. 4A), and *sod* (Fig. 4C) while inhibiting *keap* (Fig. 4B) compared with HFD fed group.

#### Inflammation-related genes expression

**Fig. 5:**
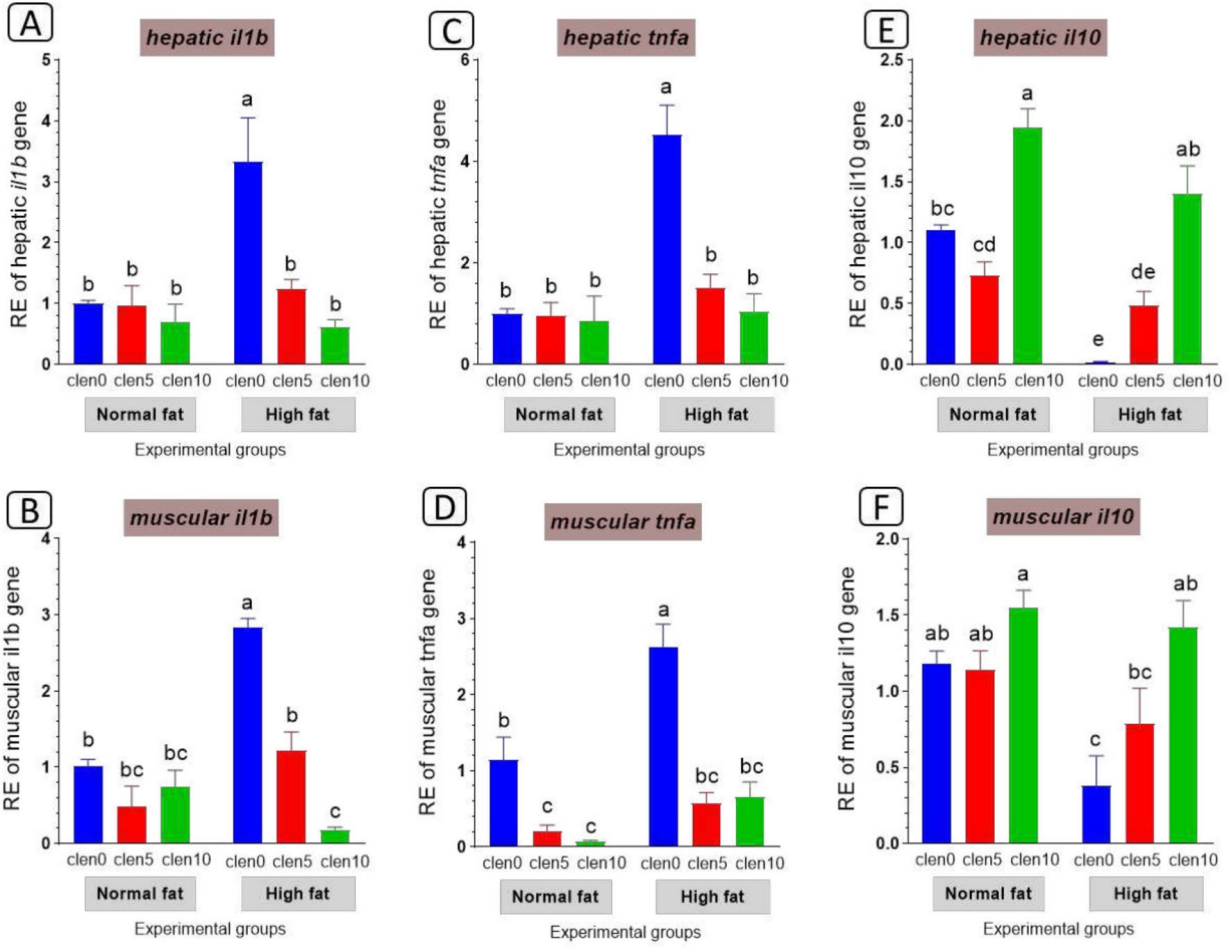
Inflammation-related genes expression in the liver and muscle tissue of Nile tilapia groups fed on normal and high fat diets with clenbuterol. il1b: Interleukin-1beta, tnfa: Tumor necrosis factor alpha; il10: Interleukin-10. Columns with different superscript letters in the same figure are significantly different (*p* ≤ 0.05).

Further molecular analysis of immune-related genes in the liver and muscle is explained in Fig. 5. HFD caused an increase in hepatic and muscular expression levels of *tnfα* (Fig. 5A), *il1b* (Fig. 5B) with reduced *il10* (Fig. 5C) compared with the normal fat-fed fish group. On the other hand clenbuterol supplementation decrease hepatic and muscular expression levels of *tnfα* (Fig. 5A), *il1b* (Fig. 5B), while upregulated *Il10* (Fig. 5C) compared with the HFD fed group.

#### Glucose metabolism-related genes

**Fig. 6:**
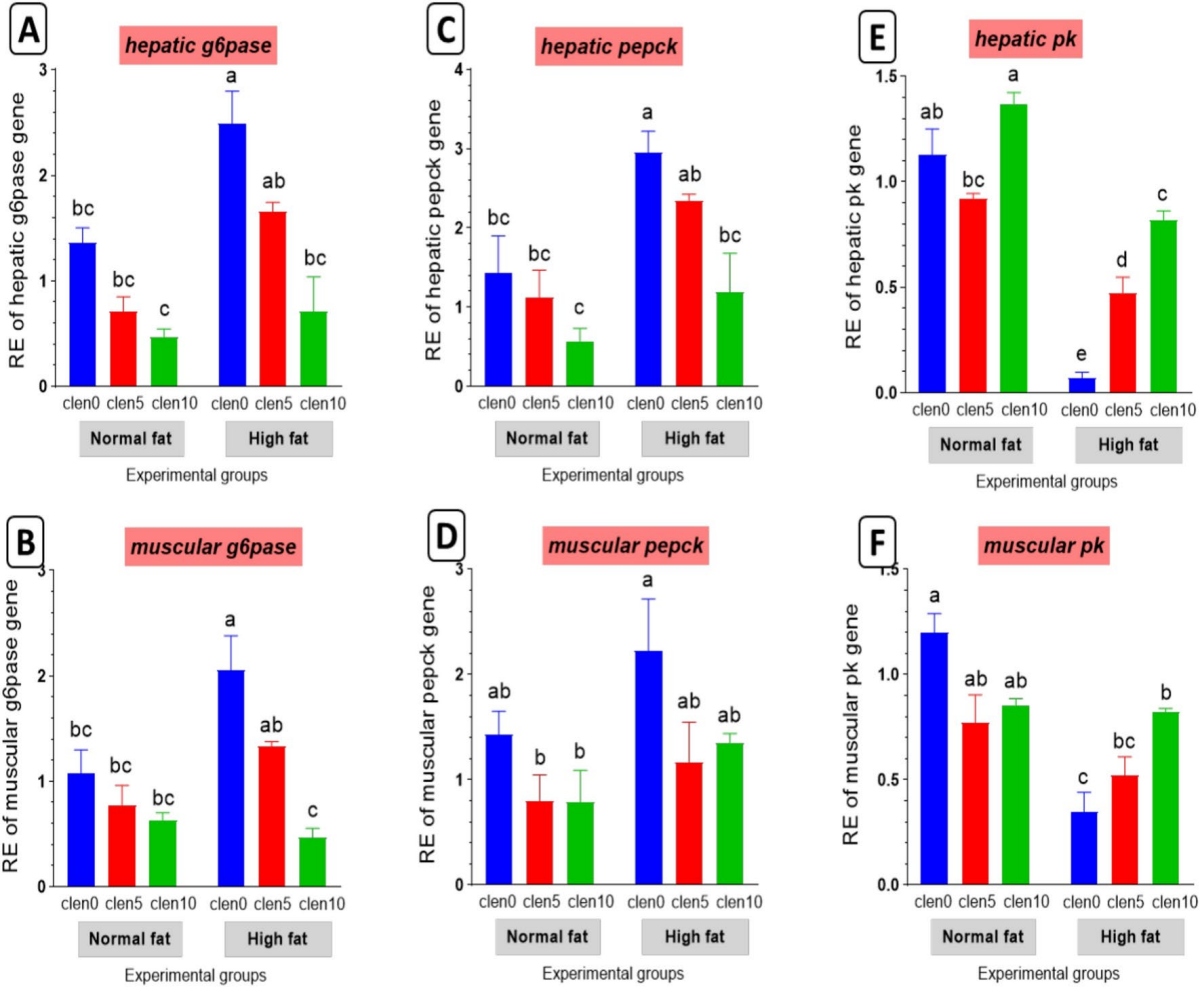
Glucose metabolism-related genes in liver and muscle of Nile tilapia groups fed on normal and high fat diets with clenbuterol. (**A** and **B**) glucose-6-phosphatase: g6pase, (**C** and D) phosphoenolpyruvate carboxy kinase: pepck, (**E** and **F**) pyruvate d kinase: pk. *P* value results of the two-way ANOVA are represented on the top of each figure. Columns with different superscript letters in the same figure are significantly different (*p* ≤ 0.05).

In the current study, clenbuterol supplementation revealed non-significant change in the expression of gluconeogenic genes *pepck*, *g6p* and *pk* matched with the control group (Fig. 6). These findings were altered in the HDF group which exhibited increased expression of gluconeogenic genes *pepck* and *g6p* with a reduced expression level of *pk*. Furthermore, these findings were reversed by clenbuterol supplementation to HFD compared with the HFD fed group.

#### Lipid metabolism-related genes

**Fig. 7:**
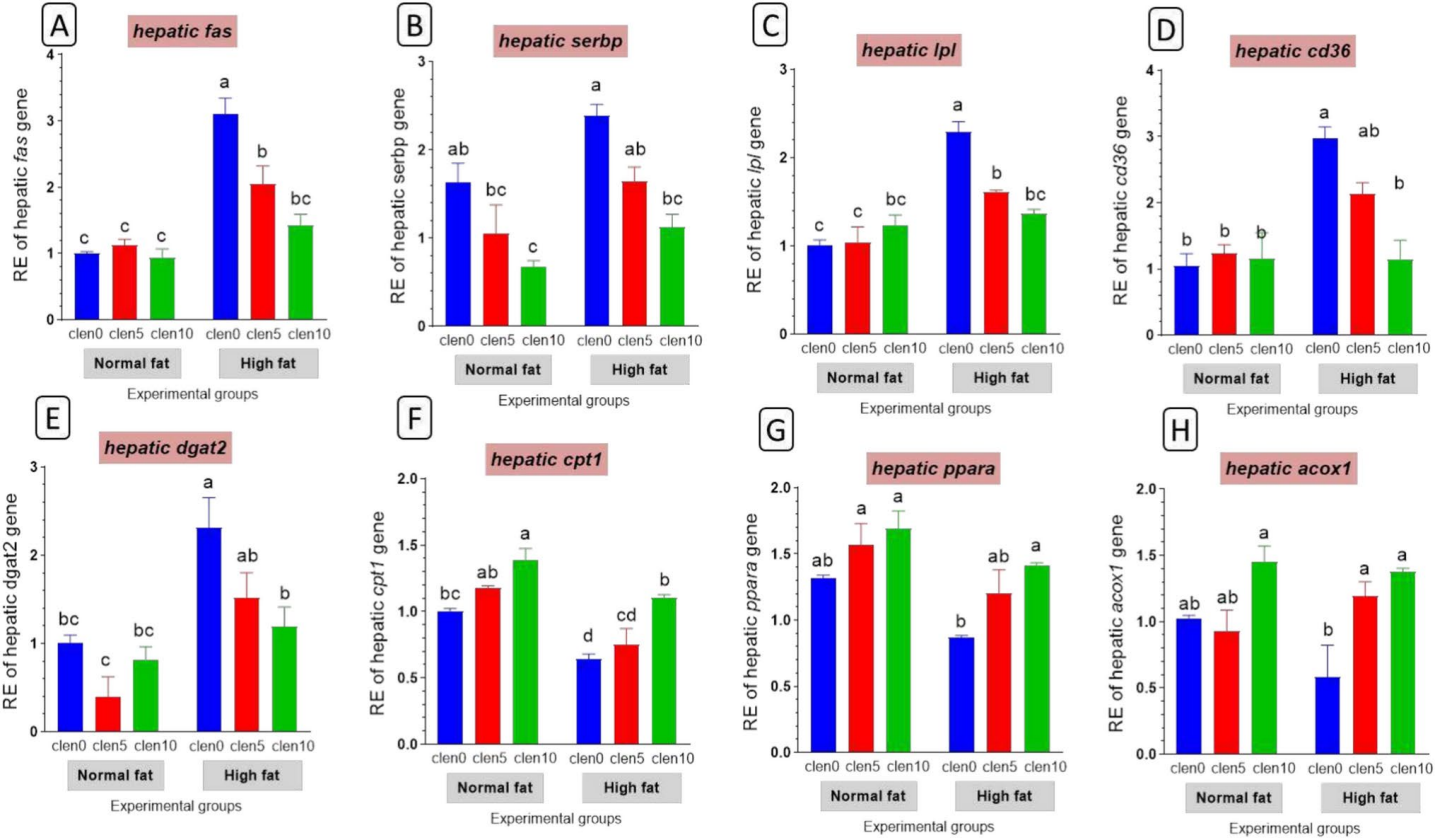
Lipid metabolism-related genes in liver of Nile tilapia groups fed on normal and high fat diets with clenbuterol. fas: fatty acid synthetase; serbp: sterol regulatory element binding protein; lpl: lipoprotein lipase; cd36: cluster of differentiation 36; dgat2: diacylglycerol O-acyltransferase 2; Cpt1: carnitine palmitoyl transferase; ppara: peroxisome proliferator-activated receptor alpha; acox1: acylcoenzyme A oxidase 1. Columns with different superscript letters in the same figure are significantly different (*p* ≤ 0.05).

High-fat diet resulted in enhanced hepatic expression levels of genes involved in lipogenesis as *fas* and *srebp-1c* (Fig. 7A,B) lipolysis (Fig. 7C), fatty acid uptake (*cd36*; Fig. 7D), TG synthesis *dgat* (Fig. 7E) import of fatty acids into mitochondria (*cpt1*; Fig. 7F), and β-oxidation *pparα* (Fig. 7G), *acox 1*(Fig. 7H).

High-fat diet caused marked mRNA expression of *fas, srebp1c, lpl, cd36* and *dgat* compared to the normal fat group, however, this increase was reserved by clenbuterol supplementation (Fig. 7A–E). Whereas carnitine palmitoyltransferase 1 (*cpt1*), peroxisome proliferator-activated receptor α (*pparα*) and acox1 expression levels were considerably diminished in fish fed HFD in comparison with the normal fat group but they were significantly upregulated by clenbuterol supplementation (Fig. 7F–H).

## Discussion

The present study has led to several important findings regarding metabolic and molecular mechanisms concerning the pathway by which clenbuterol exerts its action.

In this study, we found that a high-fat diet (HFD) significantly decreased final body weight (FBW), body weight gain (BWG), and protein efficiency ratio (PER), while increasing feed conversion ratio (FCR), intraperitoneal fat (IPF), liver fat percentage, and body fat percentage in comparison to the control group. These outcomes align with similar results observed in other marine and freshwater fish species, as largemouth bass (*Micropterus salmoides*), grass carp (*Ctenopharyngodon idella*), and giant croaker (*Nibea japonica*), where high-fat diets led to a lower final body weight gain compared to diets with normal fat levels^37,38^. Additionally, the abdominal fat and viscera somatic index were higher compared to those observed with normal fat diets^38^ also induced increment of fat deposition in the liver and abdominal cavity^5,39–41^. The current study demonstrates that clenbuterol supplementation to fish fed HFD significantly increased FBW, BWG, body protein, and ash % while reducing HSI, IPF%, liver fat, and body fat % in a dose–response manner compared to the HFD-fed fish group. Clenbuterol has been demonstrated to enhance skeletal muscle mass in mammals^42^. Additionally, Spurlock, et al.^43^ reported that clenbuterol administration stimulated anabolic activity. These findings align with prior studies in chickens, where supplementation with clenbuterol resulted in reduced weights of abdominal fat pads and elevated weights of skeletal muscles^44,45^. Similar effects were noted in rats, where clenbuterol led to reduced body fat and increased muscle mass^46,47^. Similar effects were observed in rats, with clenbuterol resulting in decreased body fat and enhanced muscle mass^48–51^. Clenbuterol-mediated skeletal hypertrophy was observed due to a marked increase in various amino acids in skeletal muscle after the administration of a glucose bolus. This observation could be explained by the preservation of predominantly glucogenic amino acids for skeletal protein synthesis when glucose is available as a fuel source^52^. It is well-known that glucose is quickly transformed into amino acids and other chemicals or metabolites in skeletal muscle^53^, and that glucose infusion dramatically enhances protein synthesis^54^.

When the intake of dietary lipids surpasses the ability of hepatic cells to oxidize fatty acids, triglyceride synthesis escalates, resulting in steatosis^55,56^. In our research, we observed a significant reduction in *fas* expression in diets supplemented with clenbuterol compared to other diets, suggesting that clenbuterol effectively inhibits excessive fatty acid synthesis induced by a high-fat diet (HFD). The liver plays a pivotal role in fatty acid metabolism. To thoroughly examine the metabolic modifications in the liver of Nile tilapia on a HFD, we analyzed several genes associated with lipid metabolism. In the liver of Nile tilapia fed a high-fat diet (HFD), the gene *pparα*, which is a key regulator of lipid metabolism, triggered the expression of multiple genes related to fatty acid β-oxidation and increased the expression of *cpt1α*^57,58^. Conversely, the *srebp1* gene, which is a crucial regulator of lipogenesis, enhanced the expression of lipogenic genes as *lpl* and *fas*^59^. Our study found that Nile tilapia fed HFD, significantly increased the expression levels of lipogenic genes (*srebp1*, *fas*, *lpl*, *dgat*, and *cd36*), while significantly decreasing lipolytic genes (*pparα*, *cpt1α*, and *acox1*) compared to those fed a normal fat diet. However, clenbuterol supplementation in HFD-fed fish reversed these findings. Similar research in other fish species, like blunt snout bream and pond loach, has demonstrated that high-fat diets lower the expression of lipolytic genes in the liver, causing fat accumulation and impairment of liver function^56–61^. Hepatic steatosis, prompted by a high-fat diet (HFD), is linked to abnormal lipid metabolism, which includes alterations in lipid synthesis, uptake, and transport^62,63^.

The insulin-sensitizing impacts of clenbuterol could be partially intervened by a reduction in hepatic lipid accumulation^64–66^. Chronic administration of clenbuterol or other β-AR agonists recovers insulin resistance^67–69^

Regarding the impact on hematological parameters, HFD resulted in a significant reduction in RBCs count, PCV, and Hb concentration, indicating microcytic hypochromic anemia (iron deficiency), while increasing WBCs, lymphocytes, and monocytic count reflects inflammatory leukogram. HFD has been shown to impact hematological parameters^70–72^ this could be attributed to various factors associated with HFD consumption, such as alterations in lipid metabolism, oxidative stress, inflammation, and insulin resistance^73^ potentially leading to conditions like anemia or impaired oxygen transport^74^. Interestingly, the dietary inclusion of clenbuterol restored the altered hematological parameters with the highest response observed with the high dose of clenbuterol. Clenbuterol’s potential to increase muscle mass and performance might indirectly affect RBC parameters. More muscle mass could mean higher oxygen demands, prompting the body to produce more red blood cells to meet these demands.

Long-term feeding of a high-fat diet (HFD) can lead to liver dysfunction, resulting in stress and potentially causing mortality in fish. In this study, liver enzyme activities were elevated in clenbuterol-treated groups compared to the control group due to the elevation of the anabolic process in muscles and the increased activity of liver enzymes intricated in manufacturing amino acids required for this process^75,76^. We observed that HFD induced elevated serum activities of ALT, AST, and LDH in conjunction with reduced total proteins, albumin, and globulins which are strongly linked with liver injury^77^ supported by our histopathological observation which revealed prominent widespread hepatic vacuolation with distinct intra-cytoplasmic vacuoles. These results are consistent with Zhang et al.^78^ who revealed considerably higher serum ALT and AST activities in the HFD group in juvenile grass carp. However, clenbuterol reversed this observation. Mohamed et al.^79,80^ reported that clenbuterol significantly increased total protein concentration by increasing protein synthesis and reducing degradation^79,80^.

Here we detected that BUN and creatinine were elevated in the HFD group supported by histopathological findings in HFD which exhibited diffuse tubular damage represented by severe tubular vacuolation and necrosis with interstitial fibrosis with mixed lymphocytes and numerous RBCs. The elevation in urea levels in serum, a catabolite of endogenous protein biomarker^81^, directly related to high amounts of hazardous nitrogen metabolites, formed during the catabolism of both proteins and amino acids, demonstrating a metabolic disorder^82^.

Concerning glucose metabolism, in the current study, clenbuterol supplementation to a normal fat diet revealed a non-significant change in blood glucose concentration (Table 6) with a non-significant change in the expression of gluconeogenic genes *pepck*, *g6p* and *pk* which catalyzes the transformation of phosphoenolpyruvate and ADP to pyruvate and ATP in glycolysis. Moreover, these findings were altered in the HFD group which showed an increase in glucose concentration and increased expression of gluconeogenic genes *pepck* and *g6p* with a reduced expression level of *pk*. These findings were inverted by clenbuterol supplementation to HFD compared with the HFD fed group (Fig. 6). Lichtenstein and Schwab^83^ suggested that individuals with higher fat intakes are more likely to develop glucose metabolism disorders, type 2 diabetes, or impaired glucose tolerance.

Gluconeogenesis is the physiological process of manufacturing glucose from non-sugar constituents, principally in the liver. However, sustained, high levels of gluconeogenesis are also a major cause of hyperglycemia in type 2 diabetes and strictly impair insulin sensitivity^84^. Here, we validate that clenbuterol can help reduce blood sugar level by regulating gluconeogenesis. This hypoglycemic effect of clenbuterol may be associated with reduced expression of *pepck* and *g6p* while enhancing *pk* implying reduced hepatic glucose output from the liver. Furthermore, decreasing hepatic glycogen levels may also reduce glucose yield from the liver, as the rate of glycogenolysis is known to be proportional to the amount of glycogen^85^. Mutually, higher insulin sensitivity and lower level of glycogen in the liver of mice treated with clenbuterol may contribute to reduced glucose output from the liver which, combined with stimulated glucose clearance in muscles, initiate restored glucose homeostasis.

Our findings indicate that HFD undesirably affected serum lipid metabolism and liver function in the HFD-fed group may point to metabolic disorders of lipids and lipoproteins as well as liver damage^86,87^. Similarly, other research have revealed significant increases in hepatic lipid content including TC, TG, LDL-C, and HDL-C in HFD-fed fish groups^88,89^. Moreover, higher levels of lipid in fish feeds can force hepatocytes to work more, potentially leading to liver damage^7^. Conversely, clenbuterol supplementation improved serum lipid profiles and hepatic enzyme activities compared to the HFD. This improvement may be attributed to lipolytic effects of clenbuterol on adipose tissue, which can confidently alter body composition by reducing TG and TC synthesis, thereby improving LDL-C, VLDL-C, and HDL-C concentrations, and promoting liver enzyme activities.

Oxidative stress is recognized as a significant factor in the advancement of liver disease caused by a HFD, resulting in mitochondrial malfunction and an inflammatory response^90^. Recent studies have emphasized that the accumulation of fat increases susceptibility to oxidative stress and compromises the antioxidant defense system in liver injury induced by a HFD^90,91^. Consistently, in this study, we observed reduced mRNA levels of antioxidant defense such as *sod* and *nrf2*, along with elevated mRNA levels of *keap1* in the liver and muscle of HFD-fed tilapia, indicating the incidence of severe oxidative damage and redox imbalance. *nrf2* is recognized as a positive regulator that protects cells against oxidative stress^92^, but severe oxidative stress can suppress the *nrf2* pathway^93^. Chambel et al.^94^ stated that activation of the *nrf2* pathway prevents lipogenesis and stimulates fatty acid synthase (*fas*) β-oxidation to protect the liver from steatosis while inactivation of the *nrf2* pathway may aggravate liver injury persuaded by hepatotoxicants^6^. Consistent with these findings, we also observed a decrease in the *nrf2* pathway in the liver and muscle of HFD-fed tilapia, suggesting that excess fat deposition impairs the *nrf2* pathway, thereby attenuating antioxidant defense.

HFD has been confirmed to stimulate lipid deposition and promoted chronic inflammation in hepatocytes of blunt snout bream fish^94–97^. Our current results suggest that HFD triggers the mRNA transcription of various inflammatory cytokines in the liver and muscle. Previous studies have found that high-fat diets can prompt inflammation by elevating the expression levels of *nf-κb*, *il-1β*, and *tnfα1* genes, worsening the inflammatory reaction^8,41^. High-fat diets have been shown to cause metabolic inflammation throughout the tissue, elevating the levels of endotoxins, circulating free fatty acids, and inflammatory mediators, resulting in low-grade systemic inflammation and distressed homeostasis in many tissues^98^. Furthermore, HFD enhanced of *nf-κb*, and inflammatory response in black seabream^19^. Similarly, *tnf-α* and *il-1β* protein levels were extraordinarily upregulated in the plasma of tilapia fed HFD matched to the control group^38,88^. Additionally, the expression levels of *nf-κb* and *il-1β* were remarkably upregulated in the gut and liver of fish fed HFD^19^. In this study, clenbuterol boosted mRNA expression of *il-10* compared with the HFD-fed group. Interleukin 10 is a critical anti-inflammatory cytokine that constrains the production of ROS and nitrogen free radicals by activating macrophages. This assistances shift the immune response from pro-inflammatory (type I) to anti-inflammatory (type II) by defeating the releasing of pro-inflammatory cytokines^99^.

The presence of inflammatory infiltrates in hepatic tissue stimulates the secretion of cytokines such as *tnf-α* and *il1b*, which contribute to the induction of insulin resistance. This metabolic disruption triggers enhanced lipolysis of TG stored in adipose tissue, leading to elevated production of fatty acids. These fatty acids counteract the anti-lipolytic effects of insulin and facilitate increased lipid uptake by the liver, resulting in dyslipidemia and hepatic steatosis^100,101^. Additionally, clenbuterol possibly will also affect insulin sensitivity by reducing inflammation^102,103^. Several β-AR agonists, including clenbuterol, have been shown to increase glucose absorption in muscle when triggered by insulin^68^. Low-dose of clenbuterol enhanced basal in vivo glucose absorption in skeletal muscle and enhanced whole-body insulin sensitivity as well as reduced hepatic steatosis under chronic stimulation of diet-induced obesity (DIO) in mice^104^.

HFD leads to fat accumulation in numerous animal species, including fish. However, storage sites of lipid in fish are extremely species-specific. For example, cod mainly store fat in the liver, whereas salmon can store great amounts of fat among muscle fibers^105.106^. Nonetheless, higher lipid levels in the liver and muscle, as well as increased mass of visceral adipose tissue, are frequently identified in most fish when fed with HFD^107,108^. However, prolonged HFD feeding eventually leads to failure in maintaining lipid homeostasis, resulting in excess lipid accumulation in non-lipid-storage tissues as skeletal muscle, liver, and heart along with intensified production of inflammatory and oxidative markers^109^. An analogous development process of HFD-induced dyslipidemia has been recorded in fish^3^. Therefore, prolonged HFD feeding in Nile tilapia is expected to result in dyslipidemic symptoms and significant changes in lipid metabolism, mostly as pathological consequences. Dietary fat has been shown to promote oxidative stress and histological abnormalities in *M. salmoides*^38^.

The link between lipid metabolism and glucose metabolism in the context of a high-fat diet involves complex interactions between substrate availability, insulin sensitivity, inflammation, mitochondrial function, and hormonal regulation. These interactions contribute to the dysregulation of glucose homeostasis observed in conditions such as obesity, type 2 diabetes, and metabolic syndrome^104^.

## Conclusion

In summary, our study demonstrated that HFD feeding induces dyslipidemia and severe liver damage, likely due to oxidative damage and inflammation. HFD impairs the Nrf2 pathway and weakens the antioxidant defense system, resulting in oxidative damage. Additionally, lipid accumulation in the liver releases pro-inflammatory factors, exacerbating liver injury. We found that clenbuterol supplementation improves growth performance and antioxidant capacity, reduces fatty acid synthesis, enhances mitochondrial β-oxidation, and improves lipid transportation in HFD-fed Nile tilapia, effectively alleviating liver fat accumulation by modulating lipid metabolism and improved glucose homeostasis most likely by stimulating glucose uptake in skeletal muscle as well as by reducing hepatic lipids.

## Supplementary Information

Supplementary Information.

## Data Availability

The authors confirm that the data supporting the findings of this study are available within the article
