# Supplementary material for: Assessing clenbuterol’s modulation of metabolic and inflammatory pathways in Nile tilapia (Oreochromas niloticous) fed high fat diet
Source: Sci Rep. 2025 Jan 10;15:1581. doi: 10.1038/s41598-024-84814-1 (PMC11724019; doi:10.1038/s41598-024-84814-1)
Supplement: Supplementary file 1 — Supplementary Information. [file 41598_2024_84814_MOESM1_ESM.pdf]

United States Department of Agriculture
Foreign Agricultural Service

GAIN
Global Agricultural Information Network

Voluntary Report – Voluntary - Public Distribution **Date:** December 03,2020

Report Number: EG2020-0056

Report Name: Egypt Endorses a Science-Based Decision on Veterinary Drugs

Country: Egypt

Post: Cairo

Report Category: Agricultural Situation, Policy and Program Announcements, Agriculture in the News, Livestock and Products

Prepared By: Shaza Omar, Senior Agricultural Specialist and Olutayo O. Akingbe, Agricultural Attaché

Approved By: Ali Abdi, Minster-Counselor for Agricultural Affairs

Report Highlights:

On November 15, National Food Safety Authority (NFSA) issued Decision No. 13/2020 setting new maximum residue limits (MRLs) for veterinary drugs including ractopamine. NFSA identified a tolerance of 20 parts per billion (ppb) for the residual of ractopamine in liver. The previous Egyptian standard regulating the import of beef liver was based on European Union Regulation Number 2377/1990, which established set a zero-tolerance level for many veterinary drug residues, including ractopamine, and became a major impediment to trade.

THIS REPORT CONTAINS ASSESSMENTS OF COMMODITY AND TRADE ISSUES MADE BY USDA STAFF AND NOT NECESSARILY STATEMENTS OF OFFICIAL U.S. GOVERNMENT POLICY

GENERAL INFORMATION

On November 15, National Food Safety Authority (NFSA) issued Decision No. 13/2020 setting new maximum residue limits (MRLs) for veterinary drugs including ractopamine. NFSA identified a tolerance level of 20 parts per billion (ppb) for the residual of ractopamine in liver, and adopted Codex MRL guideline for muscle cuts. The previous Egyptian standard regulating the import of beef liver was based on European Union Regulation Number 2377/1990, which established zero-tolerance level for many veterinary drug residues, including ractopamine. This became a major impediment to trade since 2012. The new decision of 20 ppb relaxes the zero-tolerance measure, but is lower than the Codex MRL at 40 ppb for beef liver. Decision No. 13/2020 does adopt Codex MRL for muscle cuts at 10ppb. U.S. beef exports to Egypt, which mainly consist of offal, amounted to approximately $76 million in 2019.

Decision No. 13/2020 covers two groups of veterinary drugs. The first group identifies 66 drugs and specifies accepted tolerances, most of which align with Codex recommended standards. The second group identifies 13 drugs that are prohibited on use. Article 4 of the decision articulates the requirements for the use of the veterinary drugs. The decision's annex stipulates the accepted daily intake, the chemical substance residual that is inspected, and the MRLs for different tissues according to the animal.

The decision also states that its stipulations are subject to periodic review and that NFSA will consider the results of risk assessments conducted for any of the specified veterinary drugs.

BACKGROUND INFORMATION

Egyptians consume beef liver, known locally as *kebbda*, in many local staple recipes. It is not only an affordable source of protein, but also a delicious meal for the majority of Egyptian families.

Egypt has long been one of the leading buyers of U.S. beef offal, primarily liver. The United States accounted for 82 percent of Egypt's liver imports from all sources in 2018, and 77 percent in 2019. The United States, Brazil, Australia, New Zealand and India are the major suppliers of frozen beef liver to Egypt.

Figure 1: Egypt's Main Beef Liver Suppliers

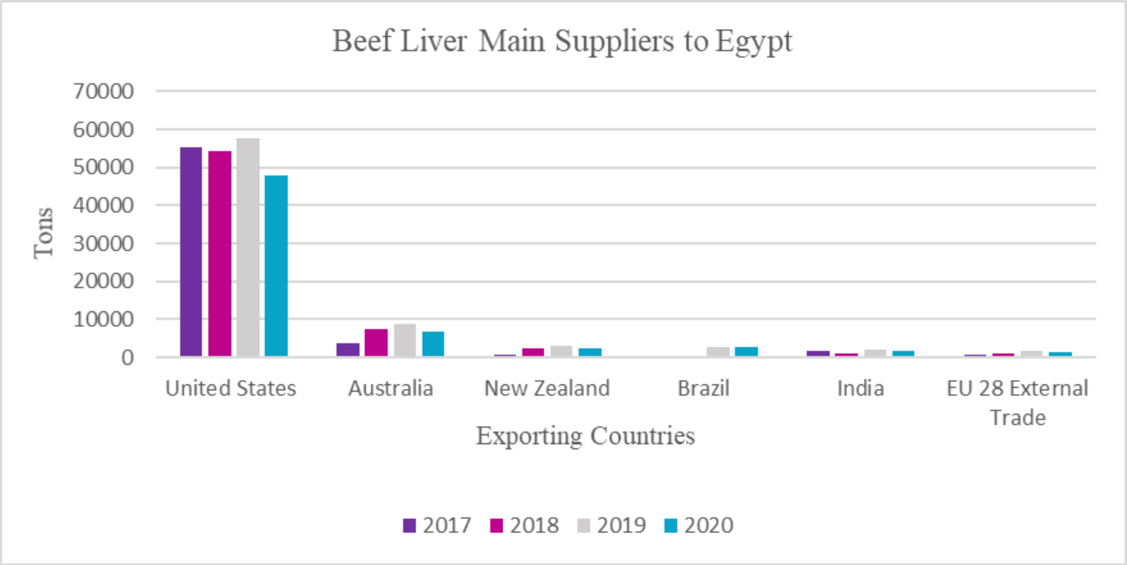

Source: TDM and U.S. Census Data
2020 figures January - October

In August 2017, Egypt's Ministry of Health (MoH) became the responsible agency for testing veterinary drug residues in beef products. Prior to that time, the Ministry of Agriculture and Land Reclamation carried out the testing, normally applying Codex standards. The MoH instituted mandatory testing of every shipment despite the fact that the vast majority of shipments cleared customs after testing. All shipments were subject to multiple rounds of testing causing long delays and costly procedures.

The Egyptian standard regulating the imports of liver are based on EU Regulation Number 2377/1990. This regulation sets a zero-tolerance level for many veterinary drug residues, including ractopamine. Ractopamine is a veterinary drug widely used in beef production. The Egyptian standard that controlled veterinary drug levels was Standard Number 7135/2010, which was implemented in 2011 by Ministerial Decree 266.

On November 15, 2019, NFSA was assigned the responsibility of food imports and announced that until its regulatory procedures are established, imports must continue to conform to existing requirements.

COST OF APPLYING ZERO-TOLERANCE

Upon an initial detection of ractopamine residue, importers requested subsequent tests on beef shipments at an additional cost. Each test required a minimum of five days, but results often take up to four weeks. Egyptian regulations require that all shipments be tested at port. The testing of each sample costs EGP 1500 ($96) and in cases where three samples are tested, sampling alone may cost up to EGP 4500 ($288). The direct costs of sampling coupled with the indirect costs associated with delayed port

clearance resulted in undue time and financial burdens that were ultimately passed on to Egyptian consumers.

DATA-DRIVEN DECISION-MAKING

Since NFSA took responsibility over food imports, it has endorsed the application of science-based decision-making. It recognizes that food safety measures should either be based on international standards or developed through assessment of risk. In the case of veterinary drugs such as ractopamine, NFSA decided to review the standard through science-based studies.

To establish the new specified tolerance, NFSA conducted a comprehensive risk assessment based on primary data collected through a questionnaire survey. The survey covered the governorates of Cairo, Giza and Qalyubia, and NFSA collected responses from 529 households alongside an additional 2,094 individuals. The purpose of the survey was to generate a daily beef liver intake index. In parallel, NFSA collected samples of liver sandwich and meals that are served in all types of restaurants and display venues. All the data were then collected and analyzed to generate a consumption pattern. The assessment concluded that a maximum residue limit of 20ppb for ractopamine in beef liver would provide an adequate protective value for Egyptian consumers with a high margin of safety.

APPENDIX I – UNOFFICIAL ENGLISH TRANSLATION

BEGIN TEXT…

The National Food Safety Authority

Decision of the Board of Directors No.13 of the Year 2020

On

the Issuance of the Technical Regulations Governing the Maximum Residue Limits for Residues of Veterinary Drugs in Foods

Board of Directors (BOD)

After reviewing the Constitution,

Law No.118 of the Year 1975 on Import and Export,

Law on the National Food Safety Authority (NFSA) enacted by Law No. 1 of the Year 2017,

Decree of the Prime Minister No. 412 of the Year 2019 on the Enactment of the Executive Regulations Implementing the Law on NFSA,

Decree of the Prime Minister No. 1296 of the Year 2020 on Re-Formation of the NFSA/BOD, and

Approval of the NFSA/BOD in the meeting held on 4 November, 2020

Decided the following:

Preamble

NFSA is an independent body aiming at protecting consumer health by ensuring that the food produced, processed, distributed or handled in the market, whether locally produced or imported from other countries, meets the highest standards of food safety. Issuing the Egyptian technical regulations by specialized technical committees is the competence of NFSA.

The present technical regulations governing the maximum residue limits (MRLs) for residues of veterinary drugs in food have been made, within the work program of technical committees (Technical Regulations Division), by NFSA in accordance with the International Standard CX/MRL 2-2018 issued and updated periodically by Codex Alimentarius Commission (CAC).

http://www.fao.org/fao-who-codexalimentarius/codex-texts/dbs/vetdrugs/veterinary-drugs/en/

Article 1

Subject Matter and Scope of Application

The present technical regulations shall pertain to MRLs for residues of veterinary drugs in foods of animal origin handled in the Arab Republic of Egypt. Such MRLs shall be based on CAC recommendations, and on risk assessment so as to ensure the appropriateness for the Egyptian conditions in case of deviations from CAC standards, as well as on the international best practices in respect of MRL estimation methods in order to ensure food hygiene with a view to protecting consumer health, and ensuring food fair trade practices.

1. **"Veterinary Drug"** shall mean any substance applied or administered to any food-producing animal, poultry, fish, and bee, whether used for therapeutic, prophylactic, or diagnostic purposes, or for modification of physiological functions or behavior.

2. **"Residues of Veterinary Drugs"** shall mean the parent compounds and/or their metabolites in any edible portion of the animal product, and include residues of associated inactive ingredients of the veterinary drug concerned.

3. "**Acceptable Daily Intake (ADI)"** shall mean an estimate by Joint Expert Committee for Food Additives (JECFA) and any Egyptian or international scientific committee approved by NFSA of the amount of a veterinary drug for the same purpose of risk assessment, expressed on a body weight basis, that can be ingested daily over a lifetime without appreciable health risk.

4. "**Maximum Residue Limit for Veterinary Drugs (MRLVD) in Foods"** shall mean the maximum concentration of residue resulting from the use of a veterinary drug and legally permitted in or on a food that can be ingested daily without being harmful to human health.

5. "**Withdrawal Time"** shall mean the period of time between the last administration of a drug to an animal and drug elimination from the body of a treated animal or residues reaching maximum residue limit (MPL), which ensures that the residues in food reach the safe levels that are not harmful to human health.

6. **"Decision Limit (CCα)"** shall mean the limit at and above which it can be concluded with an error probability of α that a sample is non-compliant.

7. **"Detection Capability (CCß)"** shall mean the smallest content of the substance that may be detected, identified and/or quantified in a sample with an error probability of β. In the case of substances for which no permitted limit has been established, the detection capability is the lowest concentration at which a method is able to detect truly contaminated samples with a statistical certainty of 1 - β. In the case of substances with an established permitted limit, this means that the detection capability is the

concentration at which the method is able to detect permitted limit concentrations with a statistical certainty of 1 - β.

8. **“Minimum Required Performance Limit (MRPL)”** shall mean minimum content of an analyte in a sample, which at least has to be detected and confirmed. It is intended to harmonise the analytical performance of methods for substances for which no permitted limit has been established.

Article 2

Supplementary References

1. The Egyptian technical regulations on sampling for estimation of MRLs permitted for residues of veterinary drugs in foods of animal origin, to be approved by NFSA, and

2. The Egyptian technical regulations on estimation methods of MRLs permitted for residues of veterinary drugs in foods of animal origin, to be approved by NFSA.

Article 3

Definitions

3.1 **“Veterinary Drug”** shall mean any substance applied or administered to any food- producing animal, poultry, fish, and bee, whether used for therapeutic, prophylactic, or diagnostic purposes, or for modification of physiological functions or behavior.

3.2 **“Residues of Veterinary Drugs”** shall mean the parent compounds and/or their metabolites in any edible portion of the animal product, and include residues of associated inactive ingredients of the veterinary drug concerned.

3.3 “**ADI”** shall mean an estimate by JECFA and any Egyptian or international scientific committee approved by NFSA of the amount of a veterinary drug for the same purpose of risk assessment, expressed on a body weight basis, that can be ingested daily over a lifetime without appreciable health risk.

3.4 “**Maximum Residue Limit for Veterinary Drugs (MRLVD) in Foods”** shall mean the maximum concentration of residue resulting from the use of a veterinary drug and legally permitted in or on a food that can be ingested daily without being harmful to human health.

3.5 “**Withdrawal Time”** shall mean the period of time between the last administration of a drug to an animal and drug elimination from the body of a treated animal or residues reaching maximum residue limit (MPL) that ensures the residues in food reach the safe levels that are not harmful to human health.

3.6 **“Decision Limit (CCα)”** shall mean the limit at and above which it can be concluded with an error probability of α that a sample is non-compliant.

3.7 **“Detection Capability (CCß)”** shall mean the smallest content of the substance that may be detected, identified and/or quantified in a sample with an error probability of β. In the case of substances for which no permitted limit has been established, the detection capability is the lowest concentration at which a method is able to detect truly contaminated samples with a statistical certainty of 1 - β. In the case of substances with an established permitted limit, this means that the detection capability is the concentration at which the method is able to detect permitted limit concentrations with a statistical certainty of 1 - β.

3.8 **“Minimum required performance limit (MRPL)”** shall mean minimum content of an analyte in a sample, which at least has to be detected and confirmed. It is intended to harmonise the analytical performance of methods for substances for which no permitted limit has been established.

Article 4
Requirements

1.The concentration of residues of veterinary drugs may not exceed MRLs provided for in Annex (1) of the present technical regulations, and the Annex shall, for each substance used as a veterinary drug, lay down the following requirements:

   a. ADI,

   b. The chemical residues of the authorized veterinary drug, to be examined, and

   c. Veterinary drug MRLs permitted in animal tissues for each animal species.

2. The Annex and the content shall, periodically and as determined by NFSA/BOD, be updated based on the international best practices, including Codex Committee on Residues of Veterinary Drugs in Foods (CCRVDF), and on studies on risk assessment so as to ensure appropriateness of some veterinary drugs at the national level in order to protect health and safety of Egyptian citizens.
3. CC-α, CC-β values and the minimum efficiency level of analysis methods for each compound shall be taken into account.
4. Residues of veterinary drugs have been divided into two groups, Group 1 consisting of the drugs with an established permitted limit in foods, Group 2 consisting of compounds for which no permitted limit of residues has been established (unpermitted) because residues of such veterinary drugs in foods pose a hazard to human health. The following tables represent the two groups:

Group 1	
Abamectin	Gentamicin
Albendazole	Imidocarb
Amoxicillin	Isometamidium
Ampicillin	Ivermectin
Avylamycin	Lasalocid sodium
Azaperone	Levamisole
Benzylpenicillin/Procaine benzylpenicillin	Lincomycin
Carazolol	Lufenuron
Ceftiofur	Melengestrol acetate
Chlortetracycline/Oxytetracycline/Tetracycline	Monensin
Clenbuterol	Monepantel
Closantel	Moxidectin
Colistin	Narasin
Cyfluthrin	Neomycin
Cyhalothrin	Nicarbazin
Cypermethrin and alpha-cypermethrin	Phoxim
Danofloxacin	Pirlimycin
Deltamethrin	Pig somatotropin

Derquantel	Progesterone
Dexamethasone	Ractopamine
Diclazuril	Sarafloxacin
Dicyclanil	Spectinomycin
Dihydrostreptomycin/Streptomycin	Spiramycin
Diminazene	Sulfadimidine
Doramectin	Teflubenzuron
Emamectin benzoate	Testosterone
Eprinomectin	Thiabendazole
Erythromycin	Tilmicosin
Estradiol-17beta	Trenbolone acetate
Febantel/Fenbendazole/Oxfendazole	Trichlorfon (Metrifonate)
Fluazuron	Triclabendazole
Flubendazole	Tylosin
Flumequine	Zeranol
**Group 2**	
Carbadox	Malachite Green
Chloramphenicol	Metronidazole
Chloropromazine	Nitrofural
Dimetridazole	Olaquindox

Furazolidone	Ronidazole
Gentian Violet	Stilbens
Ipronidazole	

Annex 1
Permitted MRLs for Residues of Veterinary Drugs in Foods in the Arab Republic of Egypt

1- ABAMECTIN (Anthelmintic Agent):

- ADI: 0-2 µg/kg body weight.
- Veterinary drug residues to be examined: (Avermectin B1a).

Animal Species	Tissue	MRL (µg/kg)	Notes
Cattle (Cow and Buffalo)	Liver	100	
Cattle (Cow and Buffalo)	Kidney	50	
Cattle (Cow and Buffalo)	Fat	100	

2- ALBENDAZOLE (Anthelmintic Agent):

- ADI: 0-50 µg/kg body weight.
- Veterinary drug residues to be examined: (2-aminosulfone metabolite) except milk, not yet identified.

Animal Species	Tissue	MRL (µg/kg)	Notes
Not specified	Meat	100	
Not specified	Liver	5000	
Not specified	Kidney	5000	
Not specified	Fat	100	
Not specified	Milk (µg/L)	100	

3- AMOXICILLIN (Antimicrobial Agent):

- ADI: 0-2 µg/kg body weight.
- Veterinary drug residues to be examined: (Amoxicillin).

Animal Species	Tissue	MRL (µg/kg)	Notes
Cattle (Cow and Buffalo)	Muscle	50	
Cattle (Cow and Buffalo)	Liver	50	

Cattle (Cow and Buffalo)	Kidney	50	
Cattle (Cow and Buffalo)	Fat	50	
Cattle (Cow and Buffalo)	Milk (µg/L)	4	
Sheep	Muscle	50	
Sheep	Liver	50	
Sheep	Kidney	50	
Sheep	Fat	50	
Sheep	Milk	4	
Pig	Muscle	50	
Pig	Liver	50	
Pig	Kidney	50	
Pig	Fat/Skin	50	
Fin Fish	Fillet	50	The term "fin fish" includes all fish species, muscle plus skin in natural proportion.
	Muscle	50	The term "fin fish" includes all fish species.

4- AMPICILLIN (Antimicrobial Agent)

- ADI: 0-3 µg/kg body weight.
- Veterinary drug residues to be examined: (Ampicillin).

Animal Species	Tissue	MRL (µg/kg)	Notes
Fin Fish	Fillet	50	The term "fin fish" includes all fish species, muscle plus skin in natural

			proportion.
	Muscle	50	The term “fin fish” includes all fish species.

5- AVILAMYCIN (Antimicrobial Agent)**:**

- ADI: 0 -2 µg/kg of body weight.
- Veterinary drug residues to be examined: Dichloroisoeverninic acid DIA.

Animal Species	Tissue	MRL (µg/kg)	Notes
Chicken	Muscle	200	
Chicken	Liver	300	
Chicken	Kidney	200	
Chicken	Fat/skin	200	
Turkey	Muscle	200	
Turkey	Liver	300	
Turkey	Kidney	200	
Turkey	Fat/skin	200	
Rabbit	Muscle	200	
Rabbit	Liver	300	
Rabbit	Kidney	200	
Rabbit	Fat/skin	200	
Pig	Muscle	200	
Pig	Liver	300	
Pig	Kidney	200	
Pig	Fat/skin	200	

6- AZAPERONE (Tranquilizing Agent):

- ADI: 0 -6 µg/kg of body weight.
- Veterinary Drug residues to be examined: Sum of Azaperol and Azaperone.

Animal Species	Tissue	MRL (µg/kg)	Notes
Pig	Muscle	60	
Pig	Liver	100	
Pig	Kidney	100	
Pig	Fat/skin	60	

7- Benzylpenicillin/Procaine Benzylpenicillin (Antimicrobial Agent) Penicillin Group:

- ADI: 30 µg/kg of body weight.
- Veterinary drug residues to be examined: Benzylpenicillin.

Animal Species	Tissue	MRL (µg/kg)	Notes
Cattle (Cow - Buffalo)	Muscle	50	
Cattle (Cow - Buffalo)	Liver	50	
Cattle (Cow - Buffalo)	Kidney	50	
Cattle (Cow - Buffalo)	Milk (µg/L)	4	
Chicken	Muscle	50	Applies to procaine benzylpenicillin only
Chicken	Liver	50	Applies to procaine benzylpenicillin only
Chicken	Kidney	50	Applies to procaine benzylpenicillin only

Animal Species	Tissue	MRL (µg/kg)	Notes
Pig	Muscle	50	
Pig	Liver	50	
Pig	Kidney	50	

8- CARAZOLOL (Beta Blocker):

- ADI: 0 -0.1 µg/kg of body weight.
- Veterinary drug residues to be examined: Carazolol.

Animal Species	Tissue	MRL (µg/kg)	Notes
Pig	Muscle	5	
Pig	Liver	25	
Pig	Kidney	25	
Pig	Fat/skin	5	

9- CEFTIOFUR (Antimicrobial Agent)

- ADI: 0 -50 µg/kg of body weight.
- Veterinary drug residues to be examined: Desfuroyl ceftiofur.

Animal Species	Tissue	MRL (µg/kg)	Notes
Cattle (Cow - Buffalo)	Muscle	1000	
Cattle (Cow - Buffalo)	Liver	2000	
Cattle (Cow - Buffalo)	Kidney	6000	

Animal Species	Tissue	MRL (µg/kg)	Notes
Cattle (Cow - Buffalo)	Fat	2000	
Cattle (Cow - Buffalo)	Milk (µg/L)	100	
Pig	Muscle	1000	
Pig	Liver	2000	
Pig	Kidney	6000	
Pig	Fat/skin	2000	

10- CHLORTETRACYCLINE/OXYTETRACYCLINE/TETRACYCLINE (Antimicrobial Agent)

- ADI: 0 -30 µg/kg of body weight.
- Veterinary drug residues to be examined: Parent drugs, singly or in combination.

Animal Species	Tissue	MRL (µg/kg)	Notes
Cattle (Cow - Buffalo)	Muscle	200	
Cattle (Cow - Buffalo)	Liver	600	
Cow	Kidney	1200	
Cow	Milk (µg/L)	100	
Fish	Muscle	200	Applies only to oxytetracycline
Giant Prawn *(Paeneus* (*monodon*	Muscle	200	Applies only to oxytetracycline
Poultry	Muscle	200	

Animal Species	Tissue	MRL (µg/kg)	Notes
Poultry	Liver	600	
Poultry	Kidney	1200	
Poultry	Eggs	400	
Sheep	Muscle	200	
Sheep	Liver	600	
Sheep	Kidney	1200	
Sheep	Milk (µg/L)	100	
Pig	Muscle	200	
Pig	Liver	600	
Pig	Kidney	1200	

11- CLENBUTEROL (Adrenoceptor Agonist):

- ADI: 0 -0.004 µg/kg of body weight.
- Veterinary drug residues to be examined: Clenbuterol.

Animal Species	Tissue	MRL (µg/kg)	Notes
Cattle (Cow - Buffalo)	Muscle	0.2	Due to the potential abuse of this drug, MRLs are recommended only when approved nationwide as a treatment for preventing preterm labor (for the purpose of relaxing the uterine wall) or as adjunctive treatment in respiratory diseases. MRL is not permitted in
Cattle (Cow - Buffalo)	Liver	0.6	
Cattle (Cow - Buffalo)	Kidney	0.6	
Cattle (Cow - Buffalo)	Fat	0.2	
Cattle (Cow -	Milk (µg/L)	0.05	

Buffalo)			Australia, New Zealand, America and Canada.<br><br>* banned in Food Animal Residue Avoidance Databank and US FDA

12- CLOSANTEL (Anthelmintic Agent):

- ADI: 0 -30 µg/kg of body weight.
- Veterinary drug residues to be examined: Closantel.

Animal Species	Tissue	MRL (µg/kg)	Notes
Cattle (Cow - Buffalo)	Muscle	1000	
Cattle (Cow - Buffalo)	Liver	1000	
Cattle (Cow - Buffalo)	Kidney	3000	
Cattle (Cow - Buffalo)	Fat	3000	
Sheep	Muscle	1500	
Sheep	Liver	1500	
Sheep	Kidney	5000	
Sheep	Fat	2000	

13- COLISTIN (Antimicrobial Agent)

- ADI: 0-7 µg/kg body weight.
- Veterinary drug residues to be examined: (Sum of colistin A and colistin B)

Animal Species	Tissue	MRL (µg/kg)	Notes
Cattle (Cow and Buffalo)	Muscle	150	
Cattle (Cow and Buffalo)	Liver	150	
Cattle (Cow and Buffalo)	Kidney	200	
Cattle (Cow and Buffalo)	Fat	150	
Cattle (Cow and Buffalo)	Milk (µg/L)	50	
Sheep	Muscle	150	
Sheep	Liver	150	
Sheep	Kidney	200	
Sheep	Fat	150	
Sheep	Milk (µg/L)	50	
Goat	Muscle	150	
Goat	Liver	150	
Goat	Kidney	200	
Goat	Fat	150	
Chicken	Muscle	150	
Chicken	Liver	150	
Chicken	Kidney	200	
Chicken	Fat	150	The MRL includes skin + fat
Chicken	eggs	300	
Turkey	Muscle	150	
Turkey	Liver	150	
Turkey	Kidney	200	

Turkey	Fat	150	The MRL includes skin + fat
Rabbit	Muscle	150	
Rabbit	Liver	150	
Rabbit	Kidney	200	
Rabbit	Fat	150	
Pig	Muscle	150	
Pig	Liver	150	
Pig	Kidney	200	
Pig	Fat	150	

14- CYFLUTHRIN (Insecticide)

- ADI: 0 -20 µg/kg of body weight.
- Veterinary drug residues to be examined: Cyfluthrin.

Animal Species	Tissue	MRL (µg/kg)	Notes
Cattle (Cow - Buffalo)	Muscle	20	
Cattle (Cow - Buffalo)	Liver	20	
Cattle (Cow - Buffalo)	Kidney	20	
Cattle (Cow - Buffalo)	Fat	200	
Cattle (Cow - Buffalo)	Milk (µg/L)	40	

15- CYHALOTHRIN (Insecticide)

- ADI: 0 -5 µg/kg of body weight.
- Veterinary drug residues to be examined: Cyhalothrin.

Animal Species	Tissue	MRL (µg/kg)	Notes
Cattle (Cow - Buffalo)	Muscle	20	
Cattle (Cow - Buffalo)	Liver	20	
Cattle (Cow - Buffalo)	Kidney	20	
Cattle (Cow - Buffalo)	Fat	400	
Cattle (Cow - Buffalo)	Milk (µg/L)	30	
Sheep	Muscle	20	
Sheep	Liver	50	
Sheep	Kidney	20	
Sheep	Fat	400	
Pig	Muscle	20	
Pig	Liver	20	
Pig	Kidney	20	
Pig	Fat	400	

16- CYPERMETHRIN AND ALPHA-CYPERMETHRIN (Insecticide)

- ADI: 0 -20 µg/kg of body weight for both forms of the drug.
- Veterinary drug residues to be examined: alpha-cypermethrin.

Animal Species	Tissue	MRL (µg/kg)	Notes

Animal Species	Tissue	MRL (μg/kg)	Notes
Cattle (Cow - Buffalo)	Muscle	50	
Cattle (Cow - Buffalo)	Liver	50	
Cattle (Cow - Buffalo)	Kidney	50	
Cattle (Cow - Buffalo)	Fat	1000	
Cattle (Cow - Buffalo)	Milk (μg/L)	100	
Sheep	Muscle	50	
Sheep	Liver	50	
Sheep	Kidney	50	
Sheep	Fat	1000	

17- DANOFLOXACIN (Antimicrobial Agent):

- ADI: 0 -20 μg/kg of body weight.
- Veterinary drug residues to be examined: Danofloxacin.

Animal Species	Tissue	MRL (μg/kg)	Notes
Cattle (Cow - Buffalo)	Muscle	200	
Cattle (Cow - Buffalo)	Liver	400	
Cattle (Cow - Buffalo)	Kidney	400	

Animal Species	Tissue	MRL (µg/kg)	Notes
Cattle (Cow - Buffalo)	Fat	100	
Chicken	Muscle	200	
Chicken	Liver	400	
Chicken	Kidney	400	
Chicken	Fat	100	Fat/skin<br><br>in normal proportions
Pig	Muscle	100	
Pig	Liver	50	
Pig	Kidney	200	
Pig	Fat	100	

18- DELTAMETHRIN (Insecticide):

- ADI: 0 -10 µg/kg of body weight.
- Veterinary drug residues to be examined: Deltamethrin**.**

Animal Species	Tissue	MRL (µg/kg)	Notes
Cattle (Cow - Buffalo)	Muscle	30	
Cattle (Cow - Buffalo)	Liver	50	
Cattle (Cow - Buffalo)	Kidney	50	
Cattle (Cow - Buffalo)	Fat	500	

Animal Species	Tissue	MRL (µg/kg)	Notes
Cattle (Cow - Buffalo)	Milk (µg/L)	30	
Chicken	Muscle	30	
Chicken	Liver	50	
Chicken	Kidney	50	Fat/skin<br>in normal proportions
Chicken	Fat	500	
Chicken	Eggs	30	
Salmon	Muscle	30	
Sheep	Muscle	30	
Sheep	Liver	50	
Sheep	Kidney	50	
Sheep	Fat	500	

19- DORAMECTIN (Anthelmintic Agent):

- ADI: 0 -1 µg/kg of body weight.
- Veterinary drug residues to be examined: Doramectin.

Animal Species	Tissue	MRL (µg/kg)	Notes
Cattle (Cow - Buffalo)	Muscle	10	There is a high concentration of drug residues at the injection site over a 35 day after subcutaneous or intramuscular administration of the drug at the recommended

Animal Species	Tissue	MRL (µg/kg)	Notes
			dose, therefore, injection site in carcasses should be excluded.
Cattle (Cow - Buffalo)	Liver	100	
Cattle (Cow - Buffalo)	Kidney	30	
Cattle (Cow - Buffalo)	Fat	150	There is a high concentration of drug residues at the injection site over a 35 day after subcutaneous or intramuscular administration of the drug at the recommended dose, therefore, injection site in carcasses should be excluded.
Cattle (Cow - Buffalo)	Milk (µg/L)	15	The use of this drug in dairy cows results in extended withdrawal periods, which should be taken into account when conducting veterinary practices
Pig	Muscle	5	
Pig	Liver	100	
Pig	Kidney	30	
Pig	Fat	150	

20- EMAMECTIN BENZOATE (Antiparasitic Agent)

- ADI: 0 -0.5 µg/kg of body weight.
- Veterinary drug residues to be examined: Emamectin B1a.

Animal Species	Tissue	MRL (µg/kg)	Notes
Salmon	Muscle	100	
Salmon	Fillet	100	Meat + skin in normal proportions
Rainbow Trout	Muscle	100	
Rainbow Trout	Fillet	100	Meat + skin in normal proportions

21- EPRINOMECTIN (Anthelmintic Agent):

- ADI: 0 -10 µg/kg of body weight.
- Veterinary drug residues to be examined: Eprinomectin B1a.

Animal Species	Tissue	MRL (µg/kg)	Notes
Cattle (Cow - Buffalo)	Muscle	100	
Cattle (Cow - Buffalo)	Liver	2000	
Cattle (Cow - Buffalo)	Kidney	300	
Cattle (Cow - Buffalo)	Fat	250	
Cattle (Cow - Buffalo)	Milk (µg/L)	20	

22- ERYTHROMYCIN (Antimicrobial Agent):

- ADI: 0 -0.7 µg/kg of body weight.
- Veterinary drug residues to be examined: Erythromycin A.

Animal Species	Tissue	MRL (µg/kg)	Notes
Poultry	Muscle	100	
Poultry	Liver	100	
Poultry	Kidney	100	
Poultry	Fat	100	The MRL includes skin + fat
Poultry	Eggs	50	
Turkey	Muscle	100	
Turkey	Liver	100	
Turkey	Kidney	100	
Turkey	Fat	100	The MRL includes skin + fat

23- DERQUANTEL (Anthelmintic Agent):

- ADI: 0 -0.3 µg/kg of body weight.
- Veterinary drug residues to be examined: Derquante.

Animal Species	Tissue	MRL (µg/kg)	Notes
Sheep	Muscle	0.3	
Sheep	Liver	0.8	
Sheep	Kidney	0.4	
Sheep	Fat	7	

24- DEXAMETHASONE (Anti-Inflammatory)

- ADI: 0 -0.015 µg/kg of body weight.
- Veterinary drug residues to be examined: Dexamethasone.

**Animal Species**	**Tissue**	**MRL (µg/kg)**	**Notes**
Cattle (Cow - Buffalo)	Muscle	1	
Cattle (Cow - Buffalo)	Liver	2	
Cattle (Cow - Buffalo)	Kidney	2	
Cattle (Cow - Buffalo)	Milk (µg/L)	0.3	
Pig	Muscle	1	
Pig	Liver	2	
Pig	Kidney	1	

25- DICLAZURIL (Antiprotozoal Agent):

- ADI: 0 -30 µg/kg of body weight.
- Veterinary drug residues to be examined: Diclazuril**.**

**Animal Species**	**Tissue**	**MRL (µg/kg)**	**Notes**
Poultry	Muscle	500	
Poultry	Liver	3000	
Poultry	Kidney	2000	
Poultry	Fat/Skin	1000	
Rabbit	Muscle	500	

Animal Species	Tissue	MRL (µg/kg)	Notes
Rabbit	Liver	3000	
Rabbit	Kidney	2000	
Rabbit	Fat	1000	
Sheep	Muscle	500	
Sheep	Liver	3000	
Sheep	Kidney	2000	
Sheep	Fat	1000	

26- DICYCLANIL (Insecticide):

- ADI: 0 -7 µg/kg of body weight.
- Veterinary drug residues to be examined: Dicyclanil**.**

Animal Species	Tissue	MRL (µg/kg)	Notes
Sheep	Muscle	150	
Sheep	Liver	125	
Sheep	Kidney	125	
Sheep	Fat	20	

27- DIHYDROSTREPTOMYCIN/STREPTOMYCIN (Antimicrobial Agent):

- ADI: 0 -50 µg/kg of body weight.
- Veterinary drug residues to be examined: Sum of dihydrostreptomycin and streptomycin.

Animal Species	Tissue	MRL (µg/kg)	Notes
Cattle (Cow -	Muscle	600	

Animal Species	Tissue	MRL (µg/kg)	Notes
Buffalo)			
Cattle (Cow - Buffalo)	Liver	600	
Cattle (Cow - Buffalo)	Kidney	1000	
Cattle (Cow - Buffalo)	Fat	600	
Cattle (Cow - Buffalo)	Milk (µg/L)	200	
Chicken	Muscle	600	
Chicken	Liver	600	
Chicken	Kidney	1000	
Chicken	Fat	600	
Sheep	Muscle	600	
Sheep	Liver	600	
Sheep	Kidney	1000	
Sheep	Fat	600	
Sheep	Milk (µg/L)	200	
Pig	Muscle	600	
Pig	Liver	600	
Pig	Kidney	1000	
Pig	Fat	600	

28- DIMINAZENE (Trypanosome):

- ADI: 0 -100 µg/kg of body weight.
- Veterinary drug residues to be examined: Diminazene.

Animal Species	Tissue	MRL (µg/kg)	Notes
Cattle (Cow - Buffalo)	Muscle	500	
Cattle (Cow - Buffalo)	Liver	12000	
Cattle (Cow - Buffalo)	Kidney	6000	
Cattle (Cow - Buffalo)	Milk (µg/L)	150	

29- ESTRADIOL-17BETA (Production Aid)

- ADI: 0 -0.05 µg/kg of body weight.
- Veterinary drug residues to be examined: Estradiol-17beta.

Animal Species	Tissue	MRL (µg/kg)	Notes
Cattle (Cow - Buffalo)	Muscle	unnecessary	Drug residues do not pose any potential hazard to human health when using the drug in accordance with good animal husbandry practice (GAHP).
Cattle (Cow - Buffalo)	Liver	unnecessary	
Cattle (Cow - Buffalo)	Kidney	unnecessary	
Cattle (Cow - Buffalo)	Fat	unnecessary	
Cattle (Cow - Buffalo)	Milk (µg/L)	unnecessary	

30- FEBANTEL/FENBENDAZOLE/OXFENDAZOLE (Anthelmintic Agent):

- ADI: 0 -7 µg/kg of body weight.
- Veterinary drug residues to be examined: Oxfendazole sulphone equivalents**.**

Animal Species	Tissue	MRL (µg/kg)	Notes
Cattle (Cow - Buffalo)	Muscle	100	
Cattle (Cow - Buffalo)	Liver	500	
Cattle (Cow - Buffalo)	Kidney	100	
Cattle (Cow - Buffalo)	Fat	100	
Cattle (Cow - Buffalo)	Milk (µg/L)	100	
Goat	Muscle	100	
Goat	Liver	500	
Goat	Kidney	100	
Goat	Fat	100	
Sheep	Muscle	100	
Sheep	Liver	500	
Sheep	Kidney	100	
Sheep	Fat	100	
Sheep	Milk (µg/L)	100	
Pig	Muscle	100	
Pig	Liver	500	
Pig	Kidney	100	

Animal Species	Tissue	MRL (µg/kg)	Notes
Pig	Fat	100	

31- FLUAZURON (Insecticide):

- ADI: 0 -40 µg/kg of body weight.
- Veterinary drug residues to be examined: Fluazuron

Animal Species	Tissue	MRL (µg/kg)	Notes
Cattle (Cow - Buffalo)	Muscle	200	
Cattle (Cow - Buffalo)	Liver	500	
Cattle (Cow - Buffalo)	Kidney	500	
Cattle (Cow - Buffalo)	Fat	7000	

32- FLUBENDAZOLE (Anthelmintic Agent):

- ADI: 0 -12 µg/kg of body weight.
- Veterinary drug residues to be examined: Flubendazole

Animal Species	Tissue	MRL (µg/kg)	Notes
Poultry	Muscle	200	
Poultry	Liver	500	
Poultry	Eggs	400	
Pig	Muscle	10	

Animal Species	Tissue	MRL (µg/kg)	Notes
Pig	Liver	10	

33- FLUMEQUINE (Antimicrobial Agent):

- ADI: 0 -30 µg/kg of body weight.
- Veterinary drug residues to be examined: Flumequine.

Animal Species	Tissue	MRL (µg/kg)	Notes
Cattle (Cow - Buffalo)	Muscle	500	
Cattle (Cow - Buffalo)	Liver	500	
Cattle (Cow - Buffalo)	Kidney	3000	
Cattle (Cow - Buffalo)	Fat	1000	
Chicken	Muscle	500	
Chicken	Liver	500	
Chicken	Kidney	3000	
Chicken	Fat	1000	
Sheep	Muscle	500	
Sheep	Liver	500	
Sheep	Kidney	3000	
Sheep	Fat	1000	
Rainbow Trout	Muscle	500	Muscle including the normal proportion of skin

Animal Species	Tissue	MRL (µg/kg)	Notes
Pig	Muscle	500	
Pig	Liver	500	
Pig	Kidney	3000	
Pig	Fat	1000	

34- GENTAMICIN (Antimicrobial Agent):

- ADI: 0 -20 µg/kg of body weight.
- Veterinary drug residues to be examined: Flumequine.

Animal Species	Tissue	MRL (µg/kg)	Notes
Cattle (Cow - Buffalo)	Muscle	100	
Cattle (Cow - Buffalo)	Liver	2000	
Cattle (Cow - Buffalo)	Kidney	5000	
Cattle (Cow - Buffalo)	Fat	100	
Cattle (Cow - Buffalo)	Milk (µg/L)	200	
Pig	Muscle	100	
Pig	Liver	2000	
Pig	Kidney	5000	
Pig	Fat	100	

35- IMIDOCARB (Antiprotozoal Agent)

- ADI: 0-10 µg/kg body weight.
- Veterinary drug residues to be examined: (Imidocarb).

Animal Species	Tissue	MRL (µg/kg)	Notes
Cattle (Cow and Buffalo)	Muscle	300	
Cattle (Cow and Buffalo)	Liver	1500	
Cattle (Cow and Buffalo)	Kidney	2000	
Cattle (Cow and Buffalo)	Fat	50	
Cattle (Cow and Buffalo)	Milk (µg/L)	50	

36- ISOMETAMIDIUM (Trypanocide)

- ADI: 0-100 µg/kg body weight.
- Veterinary drug residues to be examined: (Isometamidium).

Animal Species	Tissue	MRL (µg/kg)	Notes
Cattle (Cow and Buffalo)	Muscle	100	
Cattle (Cow and Buffalo)	Liver	500	
Cattle (Cow and Buffalo)	Kidney	1000	
Cattle (Cow and Buffalo)	Fat	100	
Cattle (Cow and Buffalo)	Milk (µg/L)	100	

37- IVERMECTIN (Anthelmintic Agent)

- ADI: 0-10 µg/kg body weight.
- Veterinary drug residues to be examined: 22,23-Dihydroavermectin B1a (H2B1a).

Animal Species	Tissue	MRL (µg/kg)	Notes
Cattle (Cow and Buffalo)	Muscle	30	

Cattle (Cow and Buffalo)	Liver	800	
Cattle (Cow and Buffalo)	Kidney	100	
Cattle (Cow and Buffalo)	Fat	400	
Cattle (Cow and Buffalo)	Milk (µg/L)	10	
Sheep	Liver	15	
Sheep	Fat	20	
Pig	Liver	15	
Pig	Fat	20	

38- LASALOCID SODIUM (Antiparasitic Agent)

- ADI: 0-5 µg/kg body weight.
- Veterinary drug residues to be examined: (Lasalocid A).

Animal Species	Tissue	MRL (µg/kg)	Notes
Chicken	Muscle	400	
Chicken	Liver	1200	
Chicken	Kidney	600	
Chicken	Skin + Fat	600	
Turkey	Muscle	400	
Turkey	Liver	1200	
Turkey	Kidney	600	
Turkey	Skin + Fat	600	
Quail	Muscle	400	
Quail	Liver	1200	
Quail	Kidney	600	

Quail	Fat/Skin	600	

39- LEVAMISOLE (Anthelmintic Agent)

- ADI: 0-6 µg/kg body weight.
- Veterinary drug residues to be examined: (Levamisole).

Species	Tissue	MRL (µg/kg)	Notes
Cattle (Cow and Buffalo)	Muscle	10	
Cattle (Cow and Buffalo)	Liver	100	
Cattle (Cow and Buffalo)	Kidney	10	
Cattle (Cow and Buffalo)	Fat	10	
Poultry	Muscle	10	
Poultry	Liver	100	
Poultry	Kidney	10	
Poultry	Fat	10	
Sheep	Muscle	10	
Sheep	Liver	100	
Sheep	Kidney	10	
Sheep	Fat	10	
Pig	Muscle	10	
Pig	Liver	100	
Pig	Kidney	10	
Pig	Fat	10	

40- LINCOMYCIN (Antimicrobial Agent):

- ADI: 0-30 µg/kg of body weight.
- Veterinary drug residues to be examined: Lincomycin.

Animal Species	Tissue	MRL (µg/kg)	Notes
Cattle (Cow-Buffalo)	Milk (µg /l)	150	
Poultry	Muscle	200	
Poultry	Liver	500	
Poultry	Kidney	500	
Poultry	Fat	100	Additional MRL for skin with adhering fat of 300 µg/kg
Pig	Muscle	200	
Pig	Liver	500	
Pig	Kidney	1500	
Pig	Fat	100	Additional MRL for skin with adhering fat of 300 µg/kg

41- LUFENURON (Pesticide) **:**

- ADI: 0-0.02 µg/kg of body weight.
- Veterinary drug residues to be examined: Lufenuron.

Animal Species	Tissue	MRL (µg/kg)	Notes

Salmon	Fillet	1350	Muscle plus skin in natural proportion
Trout	Fillet	1350	

42- MELENGESTROL ACETATE (Production Aid):

- ADI: 0-0.03 µg/kg of body weight.
- Veterinary drug residues to be examined: Melengestrol acetate.

Animal Species	Tissue	MRL (µg/kg)	Notes
Cattle (Cow-Buffalo)	Muscle	1	
Cattle (Cow-Buffalo)	Liver	10	
Cattle (Cow-Buffalo)	Kidney	2	
Cattle (Cow-Buffalo)	Fat	18	

43- MONENSIN (Antimicrobial Agent):

- ADI: 0-10 µg/kg of body weight.
- Veterinary drug residues to be examined: Monensin.

Animal Species	Tissue	MRL (µg/kg)	Notes
Cattle (Cow-Buffalo)	Muscle	10	
Cattle (Cow-Buffalo)	Liver	100	
Cattle (Cow-Buffalo)	Kidney	10	
Cattle	Fat	100	

(Cow-Buffalo)			
Cattle<br>(Cow-Buffalo)	Milk<br>(µg /l)	2	
Sheep	Muscle	10	
Sheep	Liver	20	
Sheep	Kidney	10	
Sheep	Fat	100	
Goat	Muscle	10	
Goat	Liver	20	
Goat	Kidney	10	
Goat	Fat	100	
Chicken	Muscle	10	
Chicken	Liver	10	
Chicken	Kidney	10	
Chicken	Fat	100	
Turkey	Muscle	10	
Turkey	Liver	10	
Turkey	Kidney	10	
Turkey	Fat	100	
Quail	Muscle	10	
Quail	Liver	10	
Quail	Kidney	10	
Quail	Fat	100	

44- MONEPANTEL (Anthelmintic Agent):

- ADI: 0-0.02 µg/kg of body weight.
- Veterinary drug residues to be examined: Monepantel sulfone .

Animal Species	Tissue	MRL (µg/kg)	Notes
Sheep	Muscle	500	
Sheep	Liver	7000	
Sheep	Kidney	1700	
Sheep	Fat	13000	
Cattle (Cow-Buffalo)	Fat	7000	
Cattle (Cow-Buffalo)	Kidney	1000	
Cattle (Cow-Buffalo)	Liver	2000	
Cattle (Cow-Buffalo)	Muscle	300	

45- MOXIDECTIN (Anthelmintic agent):

- ADI: 0-2 µg/kg of body weight.
- Veterinary drug residues to be examined: Moxidectin.

Animal Species	Tissue	MRL (µg/kg)	Notes
Cattle (Cow-Buffalo)	Muscle	20	Very high concentration and great variation in the level of residues at the injection site in cows over a 49 day period after

			dosing
Cattle (Cow-Buffalo)	Liver	100	
Cattle (Cow-Buffalo)	Kidney	50	
Cattle (Cow-Buffalo)	Fat	500	
Sheep	Muscle	50	
Sheep	Liver	100	
Sheep	Kidney	50	
Sheep	Fat	500	
Deer	Muscle	20	
Deer	Liver	100	
Deer	Kidney	50	
Deer	Fat	500	

46- NARASIN (Antimicrobial Agent):

- ADI: 0-5 µg/kg of body weight.
- Veterinary drug residues to be examined: Narasin A.

Animal Species	Tissue	MRL (µg/kg)	Notes

Cattle (Cow-Buffalo)	Muscle	15	
Cattle (Cow-Buffalo)	Liver	50	
Cattle (Cow-Buffalo)	Kidney	15	
Cattle (Cow-Buffalo)	Fat	50	
Chicken	Muscle	15	
Chicken	Liver	50	
Chicken	Kidney	15	
Chicken	Fat	50	
Pig	Muscle	15	
Pig	Liver	50	
Pig	Kidney	15	
Pig	Fat	50	

47- NEOMYCIN (Antimicrobial Agent):

- ADI: 0-60 µg/kg of body weight.
- Veterinary drug residues to be examined: NEOMYCIN.

Animal Species	Tissue	MRL (µg/kg)	Notes
Cattle (Cow-Buffalo)	Muscle	500	
Cattle (Cow-Buffalo)	Liver	500	

Cattle (Cow-Buffalo)	Kidney	10000	
Cattle (Cow-Buffalo)	Fat	500	
Cattle (Cow-Buffalo)	Milk (µg /l)	1500	
Chicken	Muscle	500	
Chicken	Liver	500	
Chicken	Kidney	10000	
Chicken	Fat	500	
Chicken	Eggs	1500	
Duck	Muscle	500	
Duck	Liver	500	
Duck	Kidney	10000	
Duck	Fat	500	
Goat	Muscle	500	
Goat	Liver	500	
Goat	Kidney	10000	
Goat	Fat	500	
Sheep	Muscle	500	
Sheep	Liver	500	
Sheep	Kidney	10000	
Sheep	Fat	500	
Turkey	Muscle	500	
Turkey	Liver	500	
Turkey	Kidney	10000	
Turkey	Fat	500	

Pig	Muscle	500	
Pig	Liver	500	
Pig	Kidney	10000	
Pig	Fat	500	

48- NICARBAZIN (Antiprotozoal Agent):

- ADI: 0-400 µg/kg of body weight.
- Veterinary drug residues to be examined: N,N'-bis (4-nitropheyl) urea.

Animal Species	Tissue	MRL (µg/kg)<br>µg/kg	Notes
Chicken	Muscle	200	broilers
Chicken	Liver	200	
Chicken	Kidney	200	
Chicken	Fat/skin	200	

49- PHOXIM (Insecticide):

- ADI: 0-4 µg/kg of body weight.
- Veterinary drug residues to be examined: Phoxim.

Animal Species	Tissue	MRL (µg/kg)	Notes
Goat	Muscle	50	
Goat	Liver	50	
Goat	Kidney	50	
Goat	Fat	400	
Sheep	Muscle	50	
Sheep	Liver	50	

Sheep	Kidney	50	
Sheep	Fat	400	
Pig	Muscle	50	
Pig	Liver	50	
Pig	Kidney	50	
Pig	Fat	400	

50- PIRLIMYCIN (Antimicrobial Agent):

- ADI: 0-8 µg/kg of body weight.
- Veterinary drug residues to be examined: Pirlimycin.

Animal Species	Tissue	MRL (µg/kg)	Notes
Cattle (Cow-Buffalo)	Muscle	100	
Cattle (Cow-Buffalo)	Liver	1000	
Cattle (Cow-Buffalo)	Kidney	400	
Cattle (Cow-Buffalo)	Fat	100	
Cattle (Cow-Buffalo)	Milk (µg /l)	100 µg /l	JECFA evaluated the effect of Pirlimycin residues on starter cultures and recommended an MRL of 100 µg/kg of milk. CAC members of may therefore adapt national /regional MRLs in order to address the technological aspect for trade of fresh liquid milk

			intended for processing using starter cultures.

51- PROGESTERONE (Production Aid):

- ADI: 0-30 µg/kg of body weight.
- Veterinary drug residues to be examined: Progesterone.

Animal Species	Tissue	MRL (µg/kg)	Notes
Cattle (Cow-Buffalo)	Muscle	Unnecessary	Drug residues do not pose any potential hazard to human health when using this drug in accordance with good veterinary practices.
Cattle (Cow-Buffalo)	Liver	Unnecessary	
Cattle (Cow-Buffalo)	Kidney	Unnecessary	
Cattle (Cow-Buffalo)	Fat	Unnecessary	

52- RACTOPAMINE (Production Aid):

- ADI: 0-1 µg/kg of body weight.
- Veterinary drug residues to be examined: Ractopamine.

Animal Species	Tissue	MRL (µg/kg)	Notes
Cattle (Cow-Buffalo)	Muscle	10	
Cattle (Cow-Buffalo)	Liver	20	
Cattle	Kidney	20	

(Cow-Buffalo)			
Cattle (Cow-Buffalo)	Fat	10	
Pig	Muscle	10	
Pig	Liver	20	
Pig	Kidney	20	
Pig	Fat	10	

53- SARAFLOXACIN (Antimicrobial Agent):

- ADI: 0-0.3 µg/kg of body weight.
- Veterinary drug residues to be examined: Sarafloxacin.

Animal Species	Tissue	MRL (µg/kg)	Notes
Poultry	Muscle	10	
Poultry	Liver	80	
Poultry	Kidney	80	
Poultry	Fat	20	
Turkey	Muscle	10	
Turkey	Liver	80	
Turkey	Kidney	80	
Turkey	Fat	20	

54- SPECTINOMYCIN (Antimicrobial Agent):

- ADI: 0-40 µg/kg of body weight.
- Veterinary drug residues to be examined: Spectinomycin.

Animal Species	Tissue	MRL (µg/kg)	Notes
Cattle (Cow-Buffalo)	Muscle	500	
Cattle (Cow-Buffalo)	Liver	2000	
Cattle (Cow-Buffalo)	Kidney	5000	
Cattle (Cow-Buffalo)	Fat	2000	
Cattle (Cow-Buffalo)	Milk (µg /l)	200	
Chicken	Muscle	500	
Chicken	Liver	2000	
Chicken	Kidney	5000	
Chicken	Fat	2000	
Chicken	Eggs	2000	
Sheep	Muscle	500	
Sheep	Liver	2000	
Sheep	Kidney	5000	
Sheep	Fat	2000	
Pig	Muscle	500	
Pig	Liver	2000	
Pig	Kidney	5000	
Pig	Fat	2000	

55- SPIRAMYCIN (Antimicrobial Agent):

- ADI: 0-50 µg/kg of body weight.

- Veterinary drug residues to be examined: Cattle and Chicken, sum of spiramycin and neospiramycin.

Animal Species	Tissue	MRL (µg/kg)	Notes
Cattle (Cow-Buffalo)	Muscle	200	
Cattle (Cow-Buffalo)	Liver	600	
Cattle (Cow-Buffalo)	Kidney	300	
Cattle (Cow-Buffalo)	Fat	300	
Cattle (Cow-Buffalo)	Milk (µg /l)	200	
Chicken	Muscle	200	
Chicken	Liver	600	
Chicken	Kidney	800	
Chicken	Fat	300	
Pig	Muscle	200	
Pig	Liver	600	
Pig	Kidney	300	
Pig	Fat	300	

56- SULFADIMIDINE (Antimicrobial Agent):

- ADI: 0-50 µg/kg of body weight.
- Veterinary drug residues to be examined: Sulfadimidine.

Animal Species	Tissue	MRL (µg/kg)	Notes
Cattle (Cow-Buffalo)	Milk (µg /l)	25	
not specified	Muscle	100	
not specified	Liver	100	
not specified	Kidney	100	
not specified	Fat	100	

57- TEFLUBENZURON (Pesticide):

- ADI: 0-5 µg/kg of body weight.
- Veterinary drug residues to be examined: Teflubenzuron.

Animal Species	Tissue	MRL (µg/kg)	Notes
Salmon	Muscle	400	Muscle plus skin in natural proportion
Salmon	Fillet	400	

58- TESTOSTERONE (Production Aid):

- ADI: 0-2 µg/kg of body weight.
- Veterinary drug residues to be examined: Testosterone**.**

Animal Species	Tissue	MRL (µg/kg)	Notes
Cattle (Cow-Buffalo)	Muscle	Unnecessary	Drug residues do not pose any potential hazard to human health
Cattle (Cow-Buffalo)	Liver	Unnecessary	

Cattle (Cow-Buffalo)	Kidney	Unnecessary	when using this drug in accordance with GAHP.
Cattle (Cow-Buffalo)	Fat	unnecessary	

59- THIABENDAZOLE (Anthelmintic agent):

- ADI: 0-100 µg/kg of body weight.
- Veterinary drug residues to be examined: Sum of thiabendazole and 5-hydroxythiabendazole

Animal Species	Tissue	MRL (µg/kg)	Notes
Cattle (Cow-Buffalo)	Muscle	100	The MRL also covers residues derived from feed containing the residues resulted from agricultural use.
Cattle (Cow-Buffalo)	Liver	100	
Cattle (Cow-Buffalo)	Kidney	100	
Cattle (Cow-Buffalo)	Fat	100	
Cattle (Cow-Buffalo)	Milk (µg /l)	100	
Goat	Muscle	100	
Goat	Liver	100	
Goat	Kidney	100	
Goat	Fat	100	
Goat	Milk (µg /l)	100	
Sheep	Muscle	100	
Sheep	Liver	100	
Sheep	Kidney	100	
Sheep	Fat	100	

60- TILMICOSIN (Antimicrobial Agent):

- ADI: 0-40 µg/kg of body weight.
- Veterinary drug residues to be examined: Tilmicosin.

Animal Species	Tissue	MRL (µg/kg)	Notes
Cattle (Cow-Buffalo)	Muscle	100	
Cattle (Cow-Buffalo)	Liver	1000	
Cattle (Cow-Buffalo)	Kidney	300	
Cattle (Cow-Buffalo)	Fat	100	
Chicken	Muscle	150	
Chicken	Liver	2400	
Chicken	Kidney	600	
Chicken	Fat/skin	250	
Sheep	Muscle	100	
Sheep	Liver	1000	
Sheep	Kidney	300	
Sheep	Fat	100	
Turkey	Muscle	100	
Turkey	Liver	1200	
Turkey	Kidney	1400	
Turkey	Fat/skin	250	
Pig	Muscle	100	
Pig	Liver	1500	

Pig	Kidney	1000	
Pig	Fat	100	

61- TRENBOLONE ACETATE (Growth Promoter):

- ADI: 0-0.02 µg/kg of body weight.
- Veterinary drug residues to be examined: Cattle muscle, beta-Trenbolone; Cattle Liver, alpha-Trenbolone.

Animal Species	Tissue	MRL (µg/kg)	Notes
Cattle (Cow-Buffalo)	Muscle	2	
Cattle (Cow-Buffalo)	Liver	10	

62- TRICHLORFON (Metrifonate) (Insecticide):

- ADI: 0-2 µg/kg of body weight.
- Veterinary drug residues to be examined: JECFA54 confirmed the MRL for cow's milk and the guidance levels for muscle, liver, kidney and fat of cattle recommended at the 54th WHO meeting (WHO Technical Report No. 900, 2001)

Animal Species	Tissue	MRL (µg/kg)	Notes
Cattle (Cow-Buffalo)	Milk (µg /l)	50	

63- TRICLABENDAZOLE (Anthelmintic Agent):

- ADI: 0-3 µg/kg of body weight.
- Veterinary drug residues to be examined: Ketotriclabnedazole.

Animal Species	Tissue	MRL (µg/kg)	Notes
Cattle (Cow-Buffalo)	Muscle	250	
Cattle (Cow-Buffalo)	Liver	850	
Cattle (Cow-Buffalo)	Kidney	400	
Cattle (Cow-Buffalo)	Fat	100	
Sheep	Muscle	200	
Sheep	Liver	300	
Sheep	Kidney	200	
Sheep	Fat	100	

64- TYLOSIN (Antimicrobial Agent):

- ADI: 0-30 µg/kg of body weight.
- Veterinary drug residues to be examined: Tylosin A.

Animal Species	Tissue	MRL (µg/kg)	Notes
Cattle (Cow-Buffalo)	Muscle	100	
Cattle (Cow-Buffalo)	Liver	100	
Cattle (Cow-Buffalo)	Kidney	100	
Cattle (Cow-Buffalo)	Fat	100	
Cattle	Milk (µg /l)	100	

(Cow-Buffalo)			
Chicken	Muscle	100	
Chicken	Liver	100	
Chicken	Kidney	100	
Chicken	Fat/skin	100	
Chicken	Eggs	300	
Pig	Muscle	100	
Pig	Liver	100	
Pig	Kidney	100	
Pig	Fat	100	

65- ZERANOL (Growth Promoter):

- ADI: 0-0.5 µg/kg of body weight.
- Veterinary drug residues to be examined: Zeranol.

Animal Species	Tissue	MRL (µg/kg)	Notes
Cattle (Cow-Buffalo)	Muscle	2	
Cattle (Cow-Buffalo)	Liver	10	

Unpermitted Veterinary Drug Residues in Foods

Veterinary Drug	Usage
CARBADOX	Growth Promoter
CHLORAMPHENICOL	Antimicrobial Agent
CHLORPROMAZINE	Tranquilizing Agent

DIMETRIDAZOLE	Antiparasitic Agent
FURAZOLIDONE	Antimicrobial Agent
GENTIAN VIOLET	Antifungal Agent , Antimicrobial Agent, and Anthelminthic Agent
IPRONIDAZOLE	Antiprotozoal Agent
MALACHITE GREEN	Antifungal Agent, and Antiprotozoal Agent
METRONIDAZOLE	Antiprotozoal Agent
NITROFURAL	Antimicrobial Agent
OLAQUINDOX	Antibacterial Agent
RONIDAZOLE	Antiprotozoal Agent
STILBENES	Growth Promoter

Article (5)

Technical Terms

Maximum Residue Limits (MRLs)
Veterinary Drugs
Acceptable Daily Intake (ADI)
Drug Residue
Withdrawal Periods
Anthelmintic Agent
Antimicrobial Agent
Trypanocide
Antiprotozoal Agent
Insecticide
Production Aid

(References)

1- http://www.fao.org/fao-who-codexalimentarius/standards/vetdrugs/veterinary-drugs/en
2- https://apps.who.int/food-additives-contaminants-jecfa-database/chemical.aspx?chemID=6066
3- https://eur-lex.europa.eu/legal-content/EN/TXT/?uri=CELEX:32010R0037
4- http://www.fao.org/fao-who-codexalimentarius/codex-texts/dbs/vetdrugs/veterinary-drugs/en/
5- http://www.fao.org/fileadmin/user_upload/vetdrug/docs/9-2010 -ractopamine.pdf

Article (6)

The present Decision shall be published in the Official Gazette, and shall enter into force on the day following date of publication. Any other provision contrary to what is stated in the present Decision shall be repealed.

Chairman of

Board of Directors

Dr. Hussein Mansour

Attachments:

No Attachments.
